# Improvement in Protein Domain Identification Is Reached by Breaking Consensus, with the Agreement of Many Profiles and Domain Co-occurrence

**DOI:** 10.1371/journal.pcbi.1005038

**Published:** 2016-07-29

**Authors:** Juliana Bernardes, Gerson Zaverucha, Catherine Vaquero, Alessandra Carbone

**Affiliations:** 1 Sorbonne Universités, UPMC Univ-Paris 6, CNRS, UMR 7238, Laboratoire de Biologie Computationnelle et Quantitative, Paris, France; 2 COPPE, Programa de Engenharia de Sistemas e Computação, Universidade Federal do Rio de Janeiro, Rio de Janeiro, Brazil; 3 Sorbonne Universités, UPMC Univ-Paris 6, INSERM U1135, CNRS ERL 8255, Centre d’Immunologie et des Maladies Infectieuses (CIMI-Paris), Paris, France; 4 Institut Universitaire de France, Paris, France; Stanford University, UNITED STATES

## Abstract

Traditional protein annotation methods describe known domains with probabilistic models representing consensus among homologous domain sequences. However, when relevant signals become too weak to be identified by a global consensus, attempts for annotation fail. Here we address the fundamental question of domain identification for highly divergent proteins. By using high performance computing, we demonstrate that the limits of state-of-the-art annotation methods can be bypassed. We design a new strategy based on the observation that many structural and functional protein constraints are not globally conserved through all species but might be locally conserved in separate clades. We propose a novel exploitation of the large amount of data available: 1. for each known protein domain, several probabilistic *clade-centered models* are constructed from a large and differentiated panel of homologous sequences, 2. a decision-making protocol combines outcomes obtained from multiple models, 3. a multi-criteria optimization algorithm finds the most likely protein architecture. The method is evaluated for domain and architecture prediction over several datasets and statistical testing hypotheses. Its performance is compared against HMMScan and HHblits, two widely used search methods based on sequence-profile and profile-profile comparison. Due to their closeness to actual protein sequences, clade-centered models are shown to be more specific and functionally predictive than the broadly used consensus models. Based on them, we improved annotation of *Plasmodium falciparum* protein sequences on a scale not previously possible. We successfully predict at least one domain for 72% of *P. falciparum* proteins against 63% achieved previously, corresponding to 30% of improvement over the total number of Pfam domain predictions on the whole genome. The method is applicable to any genome and opens new avenues to tackle evolutionary questions such as the reconstruction of ancient domain duplications, the reconstruction of the history of protein architectures, and the estimation of protein domain age. Website and software: http://www.lcqb.upmc.fr/CLADE.

## Author Summary

Current sequence databases contain hundreds of billions of nucleotides coding for genes and a classification of these sequences is a primary problem in genomics. A reasonable way to organize these sequences is through their predicted domains, but the identification of domains in very divergent sequences, spanning the entire phylogenetic tree of species, is a difficult problem. By generating multiple probabilistic models for a domain, describing the spread of evolutionary patterns in different phylogenetic clades, we can effectively explore domains that are likely to be coded in gene sequences. Through a machine learning approach and optimization techniques, coding for expected evolutionary constraints, we filter the many possibilities of domain identification found for a gene and propose the most likely domain architecture associated to it. The application of this novel approach to the full genome of *Plasmodium falciparum*, to a dataset of sequences from three SCOP datasets highlights the interest of exploring multiple pathways of domain evolution in the aim of extracting biological information from genomic sequences. Our new computational approach was developed with the hope of providing a novel tier of accurate and precise tools that complement existing tools such as HMMer, HHblits and PSI-BLAST, by exploring in a novel way the large amount of sequence data available. The existence of powerful databases for sequences, domains and architectures help make this hope a reality.

## Introduction

The evolutionary history of eukaryotic proteins involves rapid sequence divergence, addition and deletion of protein domains, fusion and fission of genes. This implies that protein repertoires of distantly related species differ greatly (new architectures, that is combinations of domains, are many), while domain repertoires do not (new domains are few) [1]. To account for the great diversity of domain contexts in eukaryotes, an effort was made to categorize coding regions into protein domains and domain families. An important contribution issued by this effort is the Pfam database [2], a large collection of protein families, each represented by multiple sequence alignments (MSAs) and hidden Markov models (HMMs). Pfam also provides protein domain architectures and higher-level groupings of related domains (clans). It highlighted that different architectures give rise to the diversity of proteins found in nature, and that identifying domains of a protein can provide insights into its function. Hence, the complexity of protein annotation can be simplified by focusing on domains, even though the problem of unraveling domain organization (domain architecture) still remains.

Proteins sharing more than 30% of sequence identity have a high probability also to share the same fold [3, 4]. Thus, since fold and function of a protein have generally an intimate relationship [5], strong sequence similarity is exploited by conventional alignment methods to reconstruct families of functionally related proteins and to accomplish genome annotations. Unfortunately, the complete sequencing of several organisms differing in physiology, habitat and genetics, as *Plasmodium falciparum*, brought to light the weakness of homology-based approaches to annotation [6–8]. This limitation is challenged even more by the large prokaryotic and eukaryotic metagenomic samples generated today.

Radically new homology-based annotation approaches combine information from physico-chemical properties of sequences and conserved amino acid positions in MSAs, together with sophisticated inference methods to extract a general profile (typically a profile HMM, in short pHMMs [9, 10]) of a protein domain family and use it as a signature for homology detection [8, 11–17]. This profile represents a consensus of signals characterizing a given domain in a multitude of different species. We shall speak of *sequence consensus model*, in short SCM. Conversely, other approaches [18–20] associate to each protein domain family several different profiles, built from a sample of diversified homologous sequences. The resulting set of profiles, for all protein domain families, is approximately six or seven times larger than the number of domain families, depending on the method. For instance, SUPERFAMILY [18] constructs 15 438 models for 1 962 SCOP superfamilies [21], and Gene3D [20, 22] constructs 16 933 models for 2 738 domain families. Such sets of domain families are rather small compared to the number of distinct Pfam domains (14 831 for Pfam version 27) and one would like to generate and handle multiple models representing all Pfam domains. The co-occurrence of domains within a protein was also shown to be very powerful to accurately identify domains in divergent protein sequences [23] and especially for the *P. falciparum* genome [24–27]. Nevertheless, the 37% of *P. falciparum* proteins still completely lack domain annotation and one of the main reasons is that relevant signals in sequences might become too weak to be identified by consensus if sequence divergence is too important or if the pool of sequences is biased (too small or overrepresented by certain clades). Another reason is that proteins without predicted domains might belong to novel families completely missing in Pfam and other databases.

Based on the observation that structural and functional constraints might not be globally conserved through all species, we propose a novel pipeline, called CLADE (CLoser sequences for Annotations Directed by Evolution), that identifies domains in proteins by using all known Pfam domains and the large quantity of available genomic data spanning through a large panel of species. The idea is to “decompose” the signal of consensus shared by homologous sequences, collected at the scale of the entire phylogenetic tree, into several consensus signals coming from homologous sequences collected at the scale of species within clades (we shall speak of *clade-centered models*, in short CCM), and possibly of species that are phylogenetically distant from the genome considered. To do this, we construct several profiles for each Pfam domain, starting from a large and differentiated panel of homologous sequences in a protein domain family, and we use CCMs and SCMs to search for homologous sequences in the genome to annotate. The outcomes of these models are processed and transformed into features used to train a meta-classifier, that is a Support Vector Machine (SVM) [28], that assigns a confidence score to each domain prediction. Based on this score (defined later) and on other properties, such as domain co-occurrence, CLADE finds the most probable architecture for each protein sequence by using DAMA [27], a tool that finds best domain architectures based on multi-objective optimization criteria.

By using high performance computing (HPC), CLADE demonstrates that the limits in annotation reached by current methods can be bypassed. In fact, HPC makes it possible to construct and explore a large number of profiles (a few millions) and to search, within them, for the appropriate evolutionary patterns that match the protein sequences to annotate. The idea to explore a large space of profiles and to combine the information coming from them for the prediction of domains within proteins has never been developed before and turned out to be a winning strategy that opens up new hopes and directions for the development of accurate annotation systems. In CLADE, each domain is represented by about three hundred and fifty profiles, a number that is hundreds times larger than what has been previously proposed [18–20]. In total, about 2.5 million profiles are used to annotate a genome. The information issued by these models is merged and CLADE finds agreement among models, takes into consideration their phylogenetic origin, and combines potential annotations in an accurate reconstruction of domain architectures. Both the profile-based search and the analysis of the resulting annotations require HCP. By using grid computing, we could annotate proteins lying in the twilight zone [29] of the *P. falciparum* genome, remarkably rich in A and T, and advance on the fundamental question of how to identify domains for proteins that are highly diverged. The *P. falciparum* protein annotation represents a very difficult case study. More than 44% of open reading frames in this genome remains without any putative annotation [30–32] in PlasmoDB v11.1 that reports 2 464 proteins with unknown or hypothetical function over 5 542 genes. Our methodology shows a striking improvement over current annotation approaches. No specific property of the *P. falciparum* genome, besides its localization within the Alveolata clade, is used and the method can be applied to any other genome.

We show that because of their closeness to actual protein sequences, CCMs are more specific and more functionally predictive than the broader Pfam family models, based on consensus. The accurate domain annotation reached with CCMs has important implications also for protein evolution studies. In fact, we show that CCMs provide novel information that can be exploited to explore the landscape of protein domain evolution. We shall argue that CCMs can help to trace ancient domain duplications, to reconstruct the history of protein architectures, and to better estimate protein domain age.

## Results

Our main claim is that protein domains are subject to heterogeneous evolutionary signals, possibly due to multiple evolutionary pressures of structural and functional origins. To demonstrate that this observation greatly helps to improve annotation, we first present how CLADE exploits the large quantity of available genomic information to provide protein sequence annotation. Then, we validate CLADE approach on several datasets of sequences by demonstrating that “multi-source” domain modeling is more appropriate than “mono-source” domain modeling for capturing remote homology. We compare CLADE to widely used domain prediction tools based on the mono-source approach. Finally, we report the improved annotation realized by CLADE of all *P. falciparum* proteins, known to be difficult to annotate.

### The CLADE approach

**Fig 1:**
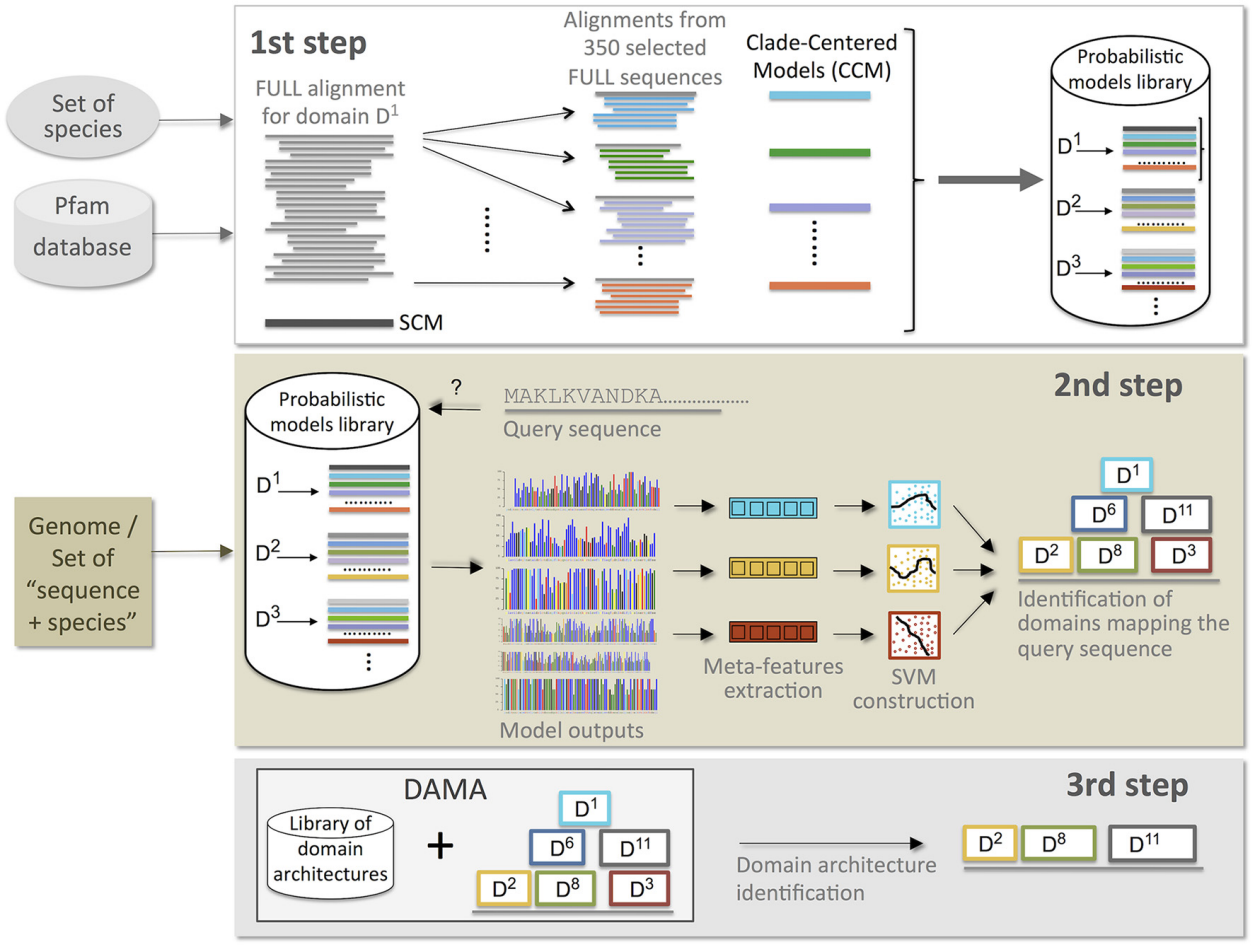
Schema representing the three main steps of CLADE method. The 1st step (**top**) concerns the construction of domain profiles from the Pfam database. A specific set of species can be furnished to CLADE (optional) to guide the selection of homologous sequences (and species) for the construction of clade-centered models (CCM), otherwise set to be a random selection. The output is a library of probabilistic models: for each Pfam domain, it contains a SCM, provided by Pfam, and a large number of CCMs, associated to the FULL set of Pfam sequences for the domain. All probabilistic models are used to identify potential domains occurring in query sequences. The schema illustrates the model construction for domain *D*^1^; it is applied to all Pfam domains. The 2nd step (**middle**) matches all models generated in step 1 against query sequences belonging to a given genome or to a set of sequences given as input, and identifies a set of potential domains occurring in the sequences. Then, it filters potential domains by using support vector machines. For each domain, it constructs a SVM that combines multiple features extracted from the SCM and CCM models associated to the domain. The schema illustrates domain identification for a given query sequence; it is applied to all input sequences. The 3rd step (**bottom**) takes the position of potential domains in a query sequence (from step 2) and runs DAMA, a tool designed to predict domain architectures from known ones.

To predict domains in protein sequences, CLADE pipeline is organized in three main steps (Fig 1). The input is a set of protein sequences coming from the same genome or from different ones. In the latter case, each sequence is accompanied by its NCBI taxon code.

#### CLADE’s first step: Construction of protein profiles to identify potential domains

In this step, CLADE uses information coming from the Pfam v27 database (Pfam_27_) and constructs probabilistic models by automatically sampling reference sequences through a large panel of species representing the whole tree of eukaryotic, archaeal, bacterial, viral worlds, and from metagenomes. If a user wants to use a preferred set of species, CLADE accepts the specified set, representing phylogenetic variability in the tree of life, as optional input.

**Fig 2:**
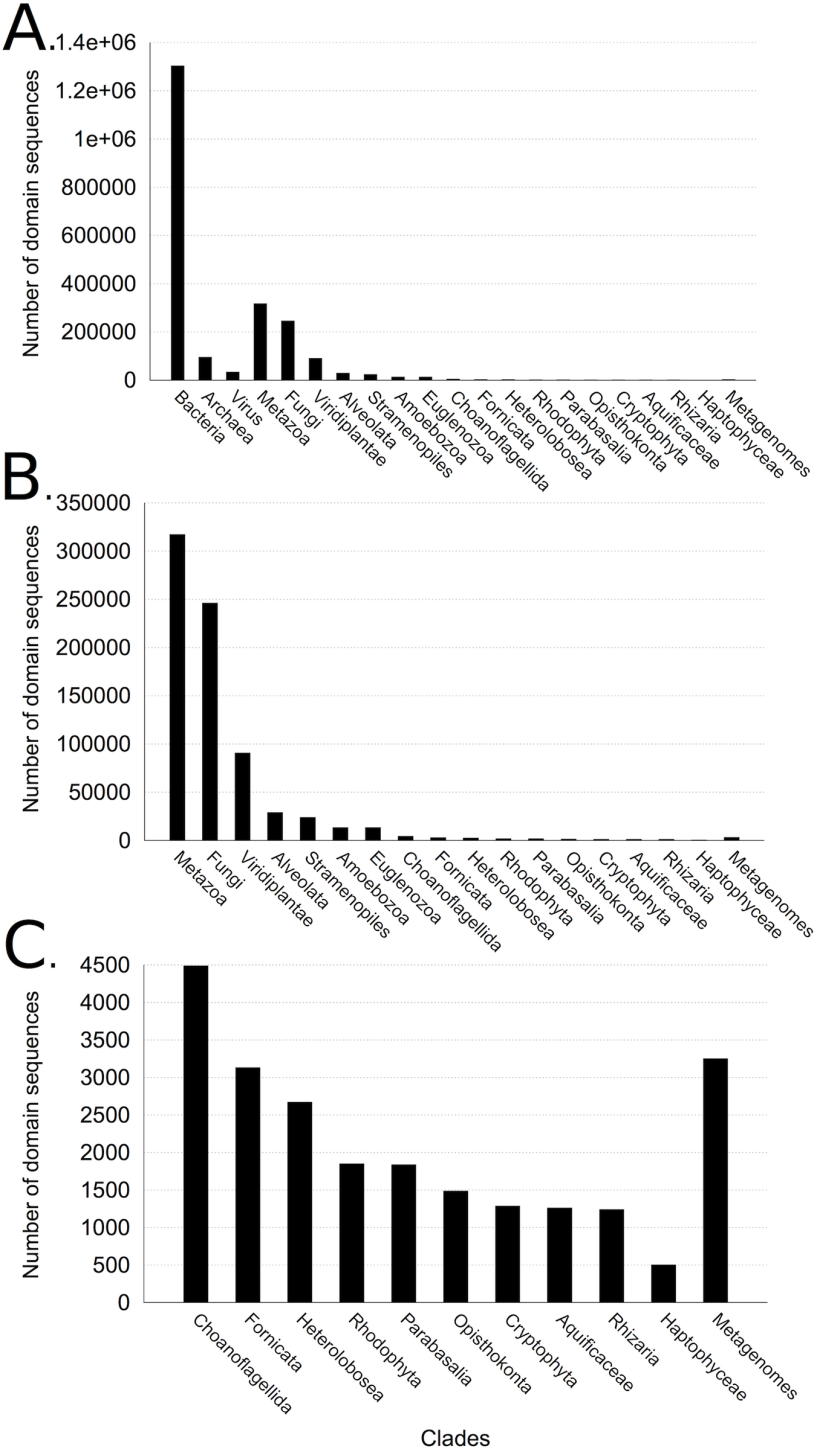
Distribution of species used for the generation of CCMs of Pfam_27_ domains. **A.** Distribution of species illustrating the spread of the organisms chosen to construct models in CLADE. Species are organized in Bacteria, Archaea, Viruses, several eukaryotic clades and environmental sequences (“Metagenomes”). For each species, at least one domain sequence has been used to construct some CCMs. “Metagenomes” are bacterial and eukaryotic sequences. **B.** Zoom over eukaryotic clades and environmental sequences appearing in A. Eukaryotic sequences are divided in 17 clades. The most represented clades are Metazoa, Fungi, Viridiplantae, Alveolata, Stramenopiles, Amoebozoa and Euglenozoa. All other clades are zoomed in **C**.

For each Pfam domain, CLADE identifies a set of homologous sequences within those in the Pfam_27_ FULL set based on their taxonomic origin. It selects species that are uniformly spread either in the phylogenetic tree of life or within the specific set of species specified by the user (see S1 Table for the set of NCBI clades considered). The selection guarantees that species belong to different phylogenetic clades and that the phylogenetic tree is well represented (see section “Clade-centered models” in Methods and S1 Fig). From each identified sequence, CLADE constructs a probabilistic profile (Fig 1, top left): it constructed 2 404 066 profiles based on the species distribution reported in Fig 2. For this, two computational approaches that underlie different evolutionary assumptions have been used. The first captures the consensus of homologous sequences. The idea behind this model is that homologous proteins should share common physico-chemical and structural features that could be described by a sequence profile based on the entire set of homologs [9]. For this reason, we call this model *sequence consensus model* (SCM). 14 831 SCMs (one for each Pfam domain) were directly downloaded from Pfam_27_. These SCMs are profile Hidden Markov Models and they have been constructed from the Pfam_27_ SEED set associated to the domain, representative of a large span of species. These models are exploited by other annotation systems, such as HMMScan [2, 33]. The second computational approach is based on a new class of models, called *clade-centered models* (CCMs), and generated 2 389 235 CCMs. This was done by taking a few hundred homologous sequences for each Pfam domain as reference sequences, and by constructing a few hundred profiles for each domain. These models span regions of protein sequence space that are not well represented in the SEED sequences from which the original Pfam SCM is constructed. CCMs might highlight motifs, structural characteristics or physico-chemical properties that are shared by a specific reference sequence and by a pool of sequences that are similar to it. Hence, if the original set of reference sequences for a domain is made of divergent homologs, CCMs are expected to describe properties that could be missed by the SCM representing global consensus. The idea behind the use of CCMs is that protein evolution pathways are bound to be few due to the numerous structural and functional constraints that a protein undergoes. This means that the evolutionary constraints that drive a protein evolution in a specific species and the corresponding (conserved, structural, physico-chemical) signals identifiable in a sequence, might be more easily detectable by looking closely at the evolutionary solutions found by some other species. We hypothesize that some species share their evolutionary solutions with the species to annotate, even if they are phylogenetically distant from it. The construction of CCMs constitutes a basic difference between CLADE approach and existing methods: CLADE uses SCMs like other methods and goes beyond them by exploiting extra information present in our databases. Namely, it constructs CCMs describing the multiple, possibly divergent, evolutionary solutions that are present in nature and uses CCMs to annotate.

#### CLADE’s second step: Multiple criteria support predictions

CLADE exploits all models produced in its first step to predict domains in the set of protein sequences to be annotated (Fig 1, middle; see section “Combining Model Predictions” in Methods and S2 Fig). It uses PSI-BLAST and HMMer (and their respective models) to screen each query sequence and identify potential domains. CLADE uses a combination of criteria, ultimately converted into a score provided by a Support Vector Machine (SVM), to specifically deal with false positives and eliminate them. This issue is fundamental in domain prediction. The SVM discriminates potential domains by evaluating which prediction is more probable among those displaying: a small E-value, a sufficiently long domain hit, the phylogenetic proximity between the taxon of the sequence to be annotated and the reference species generating the CCMs leading to annotation, and a large agreement among models leading to the prediction. Scores issued by the SVM filter boost weak domain predictions that positively satisfy several of the conditions, and penalise high confidence domains when the combination of conditions do not support the prediction. This step of merging together the results produced by different profiles for the same protein sequence, distinguishes CLADE from other multiple-profile-based approaches [18–20] (see section “Analysis of a benchmark dataset from SCOP” in Results for a comparison to the strategy previously developed by SUPERFAMILY).

#### CLADE’s third step: Protein architectures filter predictions

Domain co-occurrence is expected to enhance the level of confidence in a prediction [34, 35]. This is because: 1. the majority of proteins are multi-domain, and 2. we observe fewer domain combinations than statistically expected [34, 36, 37]. Intuitively, co-occurrence suggests functional cooperation, that is, two or more domains can interact to determine the protein function [38–40]. Once domains are selected, CLADE calls DAMA, a tool that considers domain co-occurrence and domain overlapping, and that combines several domains into most probable architectures (Fig 1, bottom).

### Multi-source versus mono-source approach to annotation

To demonstrate that “multi-source” domain modeling is more appropriate than “mono-source” domain modeling for capturing remote homology, we consider several datasets: 1. three datasets of single-domain sequences constructed from the SCOP database [21] as gold standard, 2. four datasets of randomly generated single-domain sequences satisfying different statistical hypotheses, 3. two datasets of randomly generated multi-domain sequences satisfying different statistical hypotheses. We evaluate the improvement in using an annotation that exploits multi-source domain modeling by comparing the performance of CLADE, that favors the multi-source strategy, to the performance of HMMScan and HHblits, that favor the mono-source one. We recall that HMMScan and HHblits are widely used search methods based on sequence-profile and profile-profile comparison, respectively. We estimate the false discovery rate (FDR) for the three methods. Also, with the SCOP datasets, we show that agreement among models is an important feature present in CLADE, and missing in existing multi-source domain approaches, like SUPERFAMILY. Note that CLADE skips its third step (DAMA) on datasets 1 and 2 because they comprise single-domain sequences.

#### Analysis of a benchmark dataset from SCOP

We considered three datasets of sequences coming from the database SCOP: ASTRAL95, ASTRAL30 and ASTRAL10. They are constructed from domain families sharing the same structure and they contain sequences with at most 95%, 30%, 10% of sequence identity (see section “Databases” in Methods). On these reference datasets, we tested mono-source and multi-source annotation strategies with HMMScan and HHblits supporting the mono-source strategy, and with CLADE, CLADE_*BEv*_, CLADE_HHblits and CLADE_*BEv*_HHblits supporting the multi-source strategy. CLADE_HHblits and CLADE_*BEv*_HHblits use the CLADE approach, but replace PSI-BLAST models and sequence-profile search with HHblits models and profile-profile search. They allow us to verify whether a profile-profile approach combined with multiple models improves CLADE performance. The test considers, one at the time, ASTRAL domain families and uses a leave-one-out cross validation strategy to verify how many times models constructed on all-but-one sequences help to correctly annotate left out sequences. For all tools, results are reported in Table 1; it is important to stress that the test is very stringent because it is realized at the SCOP family level. (For the details on the implementation of the test, see section “Experiment on SCOP datasets” in Methods.)

**Table 1:** Performance of CLADE, CLADE_BEv_, HMMScan and HHblits on a SCOP dataset of sequences.

**ASTRAL10**		**Mono-source**	**Multi-source**	
		**HMMScan**	**CLADE**	**CLADE**_***BEv***_
	**TP**	24	217	165
	**FP**	10	89	141
	**FN**	272	0	0
	**F-measure**	0.15	0.83	0.70
	**PPV**	0.71	0.71	0.54
	**Sen**	0.08	1	1
		**HHblits**	**CLADE_HHblits**	**CLADE**_***BEv***_HHblits
	**TP**	29	300	289
	**FP**	144	5	16
	**FN**	133	1	1
	**F-measure**	0.17	0.99	0.97
	**PPV**	0.17	0.98	0.95
	**Sen**	0.18	1	1
**ASTRAL30**		**Mono-source**	**Multi-source**	
		**HMMScan**	**CLADE**	**CLADE**_***BEv***_
	**TP**	430	994	741
	**FP**	127	229	471
	**FN**	694	28	39
	**F-measure**	0.51	0.89	0.74
	**PPV**	0.77	0.81	0.61
	**Sen**	0.38	0.97	0.95
		**HHblits**	**CLADE_HHblits**	**CLADE**_***BEv***_HHblits
	**TP**	476	1241	1150
	**FP**	502	7	97
	**FN**	318	3	4
	**F-measure**	0.54	1	0.96
	**PPV**	0.49	0.99	0.92
	**Sen**	0.6	1	1
**ASTRAL95**		**Mono-source**	**Multi-source**	
		**HMMScan**	**CLADE**	**CLADE**_***BEv***_
	**TP**	7000	8512	7772
	**FP**	710	117	857
	**FN**	923	4	4
	**F-measure**	0.9	0.99	0.95
	**PPV**	0.91	0.99	0.90
	**Sen**	0.88	1	1
		**HHblits**	**CLADE_HHblits**	**CLADE**_***BEv***_HHblits
	**TP**	3934	8537	7947
	**FP**	1679	96	686
	**FN**	3020	0	0
	**F-measure**	0.63	0.99	0.96
	**PPV**	0.7	0.99	0.92
	**Sen**	0.57	1	1

SCOP datasets ASTRAL95, ASTRAL30, ASTRAL10 contain sequences with at most 95%, 30%, 10% of sequence identity.

CLADE multi-source strategy clearly outperforms the mono-source strategy as illustrated by HMMScan and HHblits results. Note that HHblits badly handles false positives compared to HMMScan that uses a special filter for this (see section “Tools run for comparison” in Methods) and this appears clearly in the performance reported in Table 1. On the other hand, CLADE_HHblits records a clear improvement over CLADE reaching a nearly optimal performance. This highlights that the multi-source strategy successfully complements the power of HHblits. It should be noticed though, that the profile-profile search becomes extremely costly in time when expanded to a scale of hundred thousands models and thousands (or even millions, for metagenomics) of sequences to be annotated. The conversion of each query sequence into a profile, before performing profile-profile comparison, and the identification of domain specific cut-offs for all domains are highly time consuming and an approach based on a sequence-profile comparison remains more computationally reasonable.

This test allows us also to compare CLADE with SUPERFAMILY [18], a probabilistic profiles library representing all structural domains present in the SCOP database [41]. Note that CLADE has been constructed in the same spirit as SUPERFAMILY: both systems generate multiple models for a given domain, but they select among the predictions of these models with a different computational approach. SUPERFAMILY selects domains by looking at their best E-value, while CLADE exploits best E-values and also agreement among different models (second step) and domain co-existence (third step). Note that CLADE_*BEv*_ selects among different predictions of the models by using best E-values, as SUPERFAMILY does, and that domain co-existence is not tested by CLADE_*BEv*_ on the SCOP sequences, since the testing datasets contain just one domain. While CLADE and CLADE_*BEv*_ behave the same on ASTRAL95 (*F*-*measure* = *PPV* = *Sen* = 1, *PPV* = 1 for both), on more distant sequences, their performance diverges. On ASTRAL10, we observe an increasing difference for CLADE and CLADE_*BEv*_ in *F*-*measure* (0.83 and 0.7, respectively) and in *PPV* (0.71 and 0.54, respectively) at *Sen* = 1 (Table 1). This shows that, by combining results obtained by different models through SVMs, CLADE can highly improve its performance on domain detection.

To conclude, this analysis highlights that criteria other than sequence similarity play a key role in an accurate identification of protein domains. The criteria describing agreement among models and domain co-existence do not simply allow for new predictions but they provide detailed scores justifying the confidence in a prediction. They can be fruitfully used by the biologist to annotate sequences.

#### Analysis of datasets of randomly generated sequences

We performed several statistical tests to show that CLADE predicted domains are likely true positives. For this, we estimated False Discovery Rate (FDR) expressing how many times a tool, namely CLADE, HMMScan and HHblits, recognizes a known domain in a reshuffled sequence. We considered the problem of annotating sequences containing a single domain. We used four different random generations of amino-acids sequences and constructed two H0 and two H1 hypotheses. Based on the sets of sequences generated by these statistical hypotheses, we evaluated CLADE, HMMScan and HHblits. The evaluation of HHblits is included whenever the multi-source strategy is directly compared with the mono-source strategy.

As a first H0 hypothesis, we considered the set of 14 831 Pfam_27_ domain families, extracted a reference sequence from the Pfam_27_ SEED set of each domain family and generated, for each reference sequence, a random sequence by reshuffling the amino-acids (that is 1-mers) in the original one. The resulting sequence has the same length as the original one and the same amino-acids content. These random sequences were then annotated with CLADE and HMMScan and the FDR was computed for both tools. A second H0 hypothesis test was realized by considering a set of sequences representing each Pfam domain as above, and by reshuffling quadruplets of consecutive amino-acids, that is 4-mers. Again, the random sequences were annotated by CLADE and HMMScan, and the associated FDR was computed.

This test allows us to estimate how selective CLADE features are compared to the acceptance thresholds proposed by HMMScan in the identification of domains within sequences expected to contain a single domain, i.e. where co-occurrence cannot be exploited. Since CLADE uses several layers for filtering out domain predictions, its strategy is to accept more domains at the beginning and filter them based on criteria that are not used by HMMScan. But how much more does CLADE accept? The FDR computed for the two H0 hypotheses describes how permissive CLADE is at the beginning of the process, before applying DAMA, compared to HMMScan.

The two experiments were run 20 times each and 296 620 (14 831 × 20) random sequences were produced for each experiment. For each random sequence, we checked whether CLADE and HMMScan would predict a domain, and if so, whether the predicted domain was the domain originating the sequence or a different (new) one. For 1-mers, CLADE obtained a FDR of 6.98*e*-03 and HMMScan of 2*e*-04 on new domain predictions. For 4-mers, CLADE obtained a FDR of 7.6*e*-03 and HMMScan of 2*e*-04. See also S2 Table.

To show that “multi-source” domain modeling is more appropriate than “mono-source” domain modeling for capturing remote homology, we constructed two H1 hypotheses, one closer to the mono-source view, shared by the different approaches found in the literature and based on consensus sequences, and a second one closer to the multi-source view, supported by CLADE.

**Fig 3:**
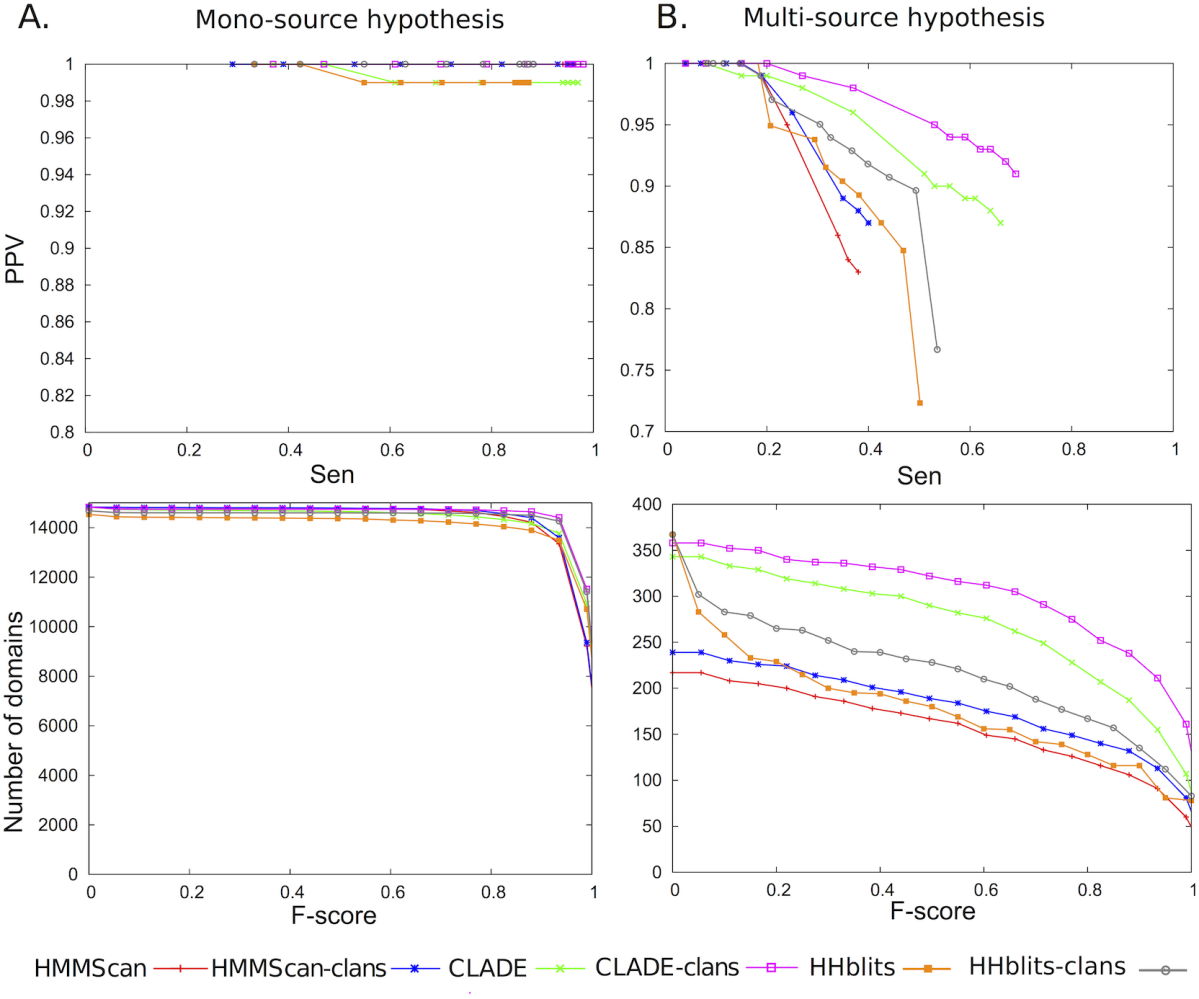
Mono-source versus multi-source hypothesis tested on randomly generated sequences. Comparison between CLADE, HMMScan and HHblits performance on the two H1 hypotheses. **A.** The mono-source hypothesis, constructed in favor of HMMScan predictions, is evaluated with a precision-recall plot (top; Sensitivity versus PPV) and by counting how many domains are discovered by the tools at a given F-score (bottom). CLADE curves are constructed by considering all domains (green) or all domains sharing the same Pfam clans (pink). The same for HMMScan (red for all domains and blue for clans) and for HHblits (orange for all domains and grey for clans) curves. **B.** The multi-source hypothesis, constructed in favor of CLADE predictions, is evaluated by two plots, described as in **A**.

The first H1 hypothesis that we explored promotes the mono-source view and it is expected to be favorably biased towards HMMScan predictions. We considered the set of 14 831 Pfam_27_ domain families and their associated SCMs, provided by Pfam_27_. Then, we generated 14 831 random sequences, each of them associated to a different probabilistic model, with HMMemit [33] (default parameters), a tool that generates artificial sequences from a probabilistic model. The generated sequences are consistent with a sequence family consensus and they are useful to test domain annotation. Next, we run HMMScan and CLADE for annotating domains in these sequences. HHblits is run by constructing profiles for each query sequence with hhblits over HHdb, the HHblits database, and by using HHsearch for a profile-profile comparison. Since the sequences come from specific domain families, we checked whether CLADE, HMMScan and HHblits could predict the right domain family for each sequence. This experiment was repeated 20 times and we evaluated sensitivity, PPV and F-score on the 296 620 generated sequences, or their associated profile. The plots describing CLADE, HMMScan and HHblits sensitivity at different PPV values, and the number of domains predicted by the three tools at different F-scores, are illustrated in Fig 3A (top and bottom). We note that for all tools, at different sensitivity values, PPV is at least 0.98. For F-scores smaller than 0.8, the number of predicted domains corresponds essentially to all domains in the set; for high F-scores, that is if both the sensitivity and the PPV are large, then the number of predicted domains remains high (about a half of all domains) for all tools. Three more curves are added to the plots: by considering Pfam_27_ clans, that is related Pfam entries that are grouped together, we observe that CLADE, HMMScan and HHblits perform comparably well and that CLADE and HHblits profit of the notion of a clan better than HMMScan. The curves plotting PPV versus sensitivity almost coincide for the three tools and they are very close when the number of domains versus F-scores are considered. This means that, when clans are not considered, CLADE predicts domains that are nevertheless very close to the original one. The performance for all systems is very good, and the plots clearly point out that all tools can easily detect domains in generated sequences when the sequence generator injects sufficiently precise information about the domain, consistently with the sequence family consensus. (Compare to the experiments based on the H0 hypotheses.) The slight advantage recorded for HMMScan is expected, since, as argued above, this H1 hypothesis is in favor of HMMScan relying on the mono-source view.

The second H1 hypothesis that we explored promotes the multi-source view and it is expected to be favorably biased towards CLADE predictions. For this, we considered the set of *P. falciparum* sequences where CLADE predicted some domain while HMMScan predicted none, and, out of it, we extracted the full set of domain sequences occurring in these *P. falciparum* sequences. Note that a *P. falciparum* sequence might contain several predicted domains, and that each of these domain sequences have been considered. We obtained a total of 571 domain sequences. For each *P. falciparum* domain sequence, we looked into the CCMs that CLADE used for predicting it and selected at most four CCMs showing an E-value < 1*e*-30, if any. Given a CCM, we considered the sequence originally used to construct it and produced a pHMM with jackHMMer [33]. This means that we have at most 4 CCMs models associated to each one of the 571 domain sequences. For each sequence, we picked one of their associated CCMs with a uniform probability and generated a sequence from it with HMMemit. The generation of sequences was performed 20 times. For each resulting sequence, we constructed a profile to evaluate HHblits. We annotated these 11 420 generated sequences or profiles with CLADE, HMMScan and HHblits, and evaluated the three tools with sensibility, PPV and F-score as before.

In the two plots of Fig 3B (top and bottom), we observe that CLADE performs better than HMMScan and HHblits. This remains true when a more permissive evaluation is realized at the clan level rather than the usual family level. For all methods, the clan evaluation shows that many false predictions are domains that belong to the same clan of the true domain. We also observe that HHblits outperformed HMMScan, the use of profile-profile searching being more efficient [17, 42]. This highlights the interest of using multiple models in domain identification. Nevertheless, we observe that HMMScan performance is very good, considering that it did not find the domain on the original *P. falciparum* sequence. In this respect, it should be noticed that:

1. here the task is somewhat easier, since HMMScan is asked to annotate a domain sequence and not a larger *P. falciparum* sequence where the position of the domain has to be identified.
2. we constructed CCMs starting from specific sequences from which CLADE annotated the corresponding *P. falciparum* domain. They are possibly heterogeneous and they are supposed to guide HMMScan domain recognition, since we know from CLADE analysis that their reference sequence can help annotation. Yet, the lower HMMScan performance demonstrates that the combination of several CCMs (multiple-source modeling) is important for annotation. HMMScan does not use the idea of agreement between heterogeneous models while CLADE does.

In conclusion, CLADE, HMMScan and HHblits predict more easily domains in the first H1 dataset than in the second one (see plots on the bottom of Fig 3A and 3B, where the *y*-axes have not the same scale). By comparing the plots on the top of Fig 3A and 3B, we note that CLADE performs similarly to HMMScan and HHblits on the first dataset, while on the second one, constructed from a list of hard examples of remote homology, the difference among the three systems is much more important. The heterogeneity of the domain models appears crucial for obtaining an improved performance (considering clans or not) and a successful remote homology detection. Clearly, HHblits profile-profile comparison improves the mono-source strategy performance of HMMScan, but it remains poor when compared to CLADE multi-source strategy.

#### CLADE FDR computed on multi-domain sequences

**Fig 4:**
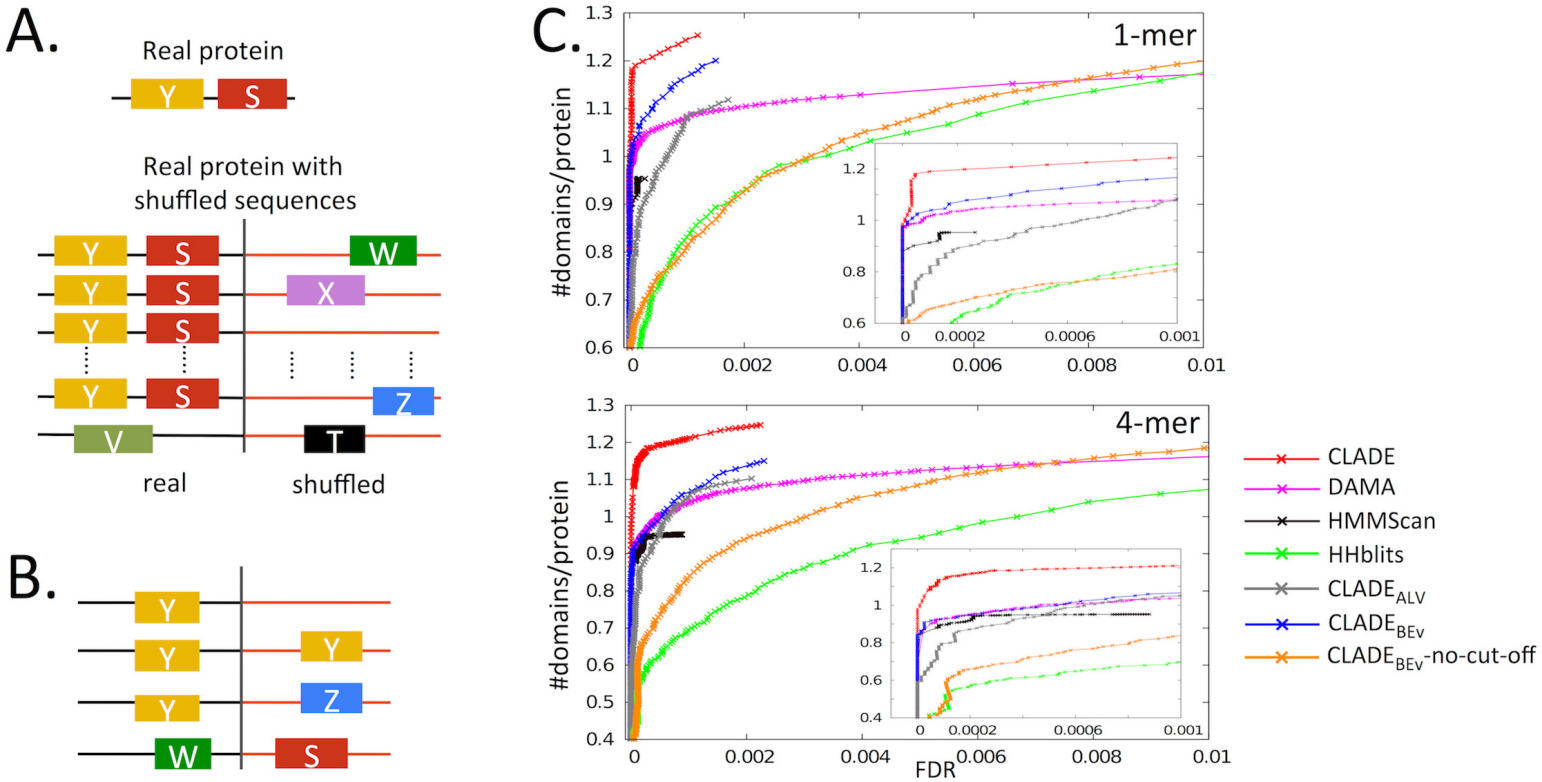
CLADE predicts more domains over a range of FDRs. **A.** Illustration of the FDR estimation procedure. For each original protein sequence, we make predictions on it and on twenty shuffled sequences concatenated to the original sequence, to allow “real” domains (Y, S) to boost false predictions on the shuffled sequence (domains V, Z, X) when using domain co-occurrence. The estimated FDR is the ratio of false predictions per protein to the total number of predictions per protein. Notice that domain co-occurrence might imply the original protein sequence to be re-annotated (see domain V in the bottom sequence of the drawing). **B.** Illustration of the four types of annotation that can be induced by the concatenation with a shuffled sequence discussed in the text. **C.** The y-axis is the number of predicted domains per protein (“signal”), while the x-axis is the FDR (“noise”), so better performing methods have higher curves (more signal for a given noise threshold). CLADE (red) outperforms HMMScan (black), HHblits (green) and DAMA (pink) on the two datasets, 1-mer (top) and 4-mer (bottom), obtained by randomly reshuffling *P. falciparum* sequences (see text). CLADE has been tested under several restrictions and the resulting FDR curves have been added to the plot: CLADE_*ALV*_ (grey), CLADE_*BEv*_ (blue) and CLADE_*BEv*_-no-cut-off (orange). The inset plot zooms the curves on small FDR values (< 0.001).

We estimated CLADE FDR on the prediction of multi-domain proteins. To do so, we consider a FDR strategy that is based on the first H0 hypothesis introduced above, generating random sequences that preserve the same amino-acids composition of the original sequences. Here, we generated random sequences starting from the 5 542 *P. falciparum* sequences. For each of these sequences, we concatenated the real sequence to its random reshuffling (Fig 4A). Then, to annotate the domains and evaluate the performance of the systems with an estimation of their FDR, we run CLADE and HMMScan on this dataset of sequences and HHblits on the associated profiles. Several situations may arise, as illustrated in Fig 4B: 1. the original sequence is annotated as before, and no annotation is found in the reshuffled one; 2. the reshuffled sequence as well as the original sequence are both annotated with the original domain annotation; 3. the original domain annotation is found in the original sequence and a different annotation is found in the reshuffled one; 4. both subsequences are newly annotated. With respect to these four scenarios, we counted as false positives all outcomes of kind 2, 3 and 4. That is, we just check whether the random sequence can be annotated at all.

We repeated the construction 20 times and considered as FDR, the average FDR of the 20 experiments. We also checked the second H0 hypothesis introduced above, based on the reshuffling of 4-mers, following the same procedure as for 1-mers. The idea behind this model is that 4-mers within a protein sequence might be more likely to occur than others, since protein sequences might contain repetitive patterns.

A comparison among the tools is reported in Fig 4C (top) (see section “FDR curves” in Methods and S3 Fig), where CLADE is shown to predict a much higher number of domains than HMMScan and HHblits for any fixed noise threshold (that is, the same FDR) for both the 1-mer and the 4-mer reshuffling. HMMScan and CLADE predict a comparable small number of domains in the reshuffled parts, while CLADE predicts more domains than HMMScan on the real parts. In contrast, HHblits performance is highly influenced by two main factors. First, the construction of a profile for the shuffled part of the query sequence introduces extra noise in the evaluation of HHblits, contrary to HMMScan and CLADE that directly work on sequences. This factor seems to particularly affect HHblits in 4-mers, sequences that are harder to analyze than 1-mers. Second, the lack of domain specific cut-off acceptance for HHblits, contrary to HMMScan and CLADE that filter out a large number of false positives due to suitable domain specific cut-offs. Note that such cut-offs are crucial for CLADE. In fact, when we test CLADE_*BEv*_-no-cut-off, that is the version of CLADE that does not include the SVM filter, that does not use domain specific cut-offs and that considers a score system based on best E-values only, the behavior of the system is essentially identical to the one of HHblits on 1-mer as illustrated in Fig 4. Compare it with the curve associated to CLADE_*BEv*_ also. (See curves on the 4-mer plot as well.)

In conclusion, we started from random sequences, relatively far from real protein sequences, and showed that CLADE has a significantly different behavior from HMMScan and HHblits on these datasets. Notice that the two H0 hypotheses would ask for the three tools to minimize the number of predictions in random sequences. Even if HMMScan and CLADE detect some domain, their performances remain very good as shown by the plots. HHblits’ lack of filtering for false positives explains its poorer performance. It is interesting to notice that DAMA [27], the tool used in CLADE 3rd step for reconstructing the best architecture from domain hits, performs better than HMMScan based on E-value, when it is evaluated on hits obtained with SCMs, as reported in Fig 4C. The CLADE curve evaluates how much the usage of CCMs improves annotation compared to the usage of SCMs only. Note that DAMA is today the state-of-the-art method compared to tools handling domain co-occurrence such as MDA [23], CODD [24] and dPUC [26]. The curves demonstrate that, by combining DAMA with clade-centered models, domain annotation highly improves.

Note that Fig 4C reports the behavior of CLADE when the model library is restricted to CCMs from the Alveolata clade only (CLADE_*ALV*_). One observes that at the same FDR value, the number of predictions is much lower for CLADE_*ALV*_ than for CLADE.

### Analysis of the dataset of all *P. falciparum* proteins

Upheld by CLADE performance on the SCOP benchmark datasets and the FDR tests, we checked whether CLADE could significantly contribute to the identification of domains within the full set of *P. falciparum* proteins. A large number (2464) of *P. falciparum* proteins has no identified domain in PlasmoDB and it remains with no domain identification (2068) even after the analysis of existing *in silico* predictive methods [2, 24, 26]. We performed a large scale domain prediction and compared CLADE results against annotations obtained with HMMScan.

All evaluations reported below were realized with the same CLADE parameters (with an E-value cut-off at 1*e*-3 and by adopting a specific SVM probability cut-off for each domain). See section “CLADE pipeline, parameter settings and tools used in CLADE” in Methods.

#### Domain annotation of all *P. falciparum* proteins

**Fig 5:**
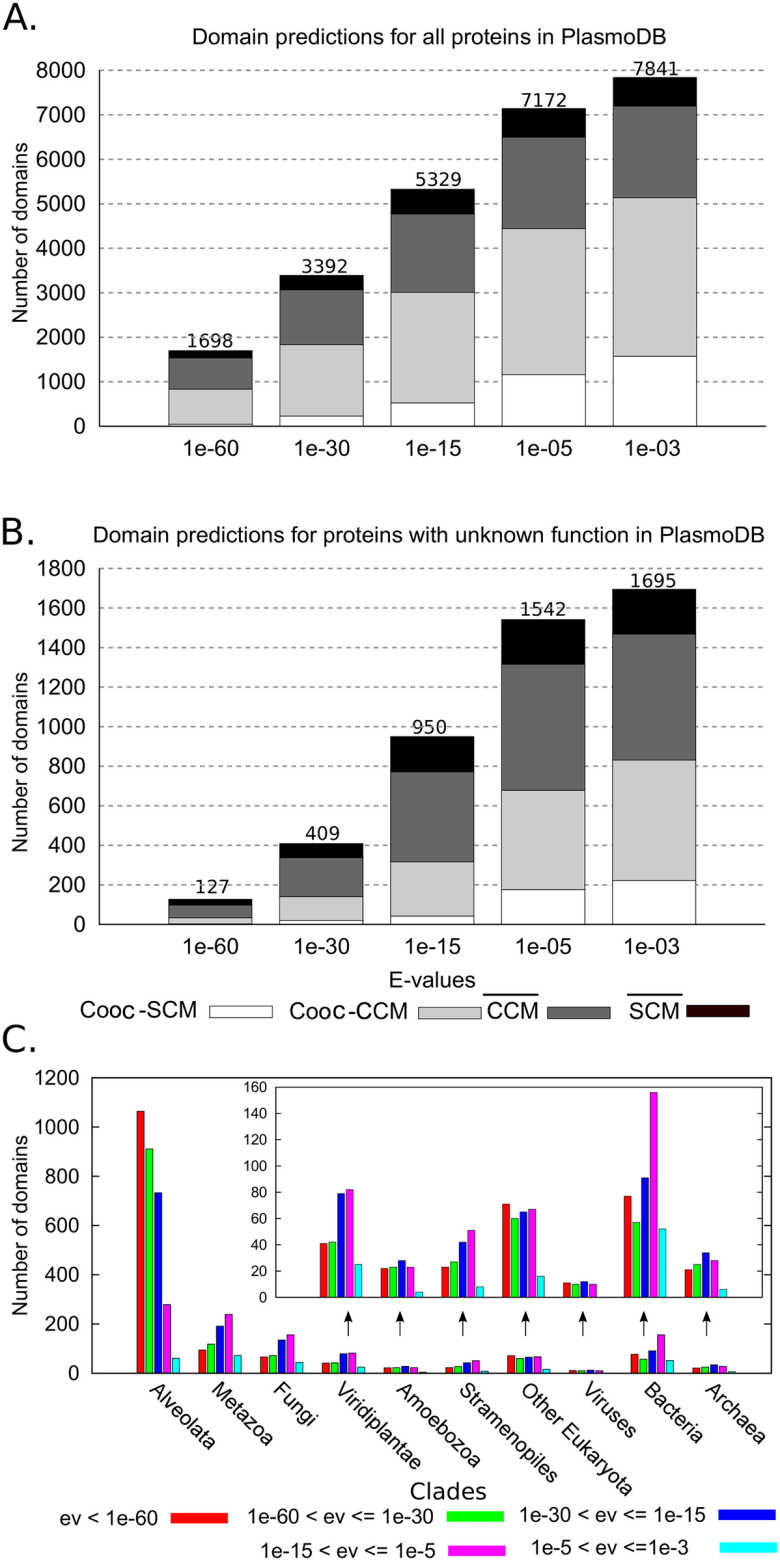
Domain predictions for *P. falciparum* proteins. **A.** Cumulative plot of domain predictions, for all *P. falciparum* proteins in PlasmoDB. All domains identified with an E-value ≤ *E* are counted, where *E* indexes the bins. The height of a bin corresponds to the total number of predictions. Predicted domains are classified in two groups, depending on whether they have been predicted with (white and light grey) or without (dark grey and black) domain co-occurrence, and two subgroups, depending on whether they have been identified through SCMs (white and black; Cooc-SCM and $\bar{\text{SCM}}$) or by means of CCMs (light and dark grey; Cooc-CCM and $\bar{\text{CCM}}$). Note that $\text{Cooc-CCM}\cup \bar{\text{CCM}}$ equals the set of domains detected by CCMs, and that $\text{Cooc-SCM}\cup \bar{\text{SCM}}$ is the set of domains detected by SCMs. **B.** Cumulative plot of domain predictions for proteins with “unknown function” in PlasmoDB. Here, we consider that a domain has “unknown function” if it is annotated in PlasmoDB with one of the following labels: unknown function, product unspecified, hypothetical protein, pseudogene and conserved *P. falciparum* protein family. Bins are as in **A**. **C.** Distribution of species generating all CCMs used to annotate the *P. falciparum* genome. The inset plot is a zoom of the last seven clades; arrows help to identify names of clades. (Also, see S4 Fig for the distribution of species generating CCMs used to detect new domains.)

Over the 5 542 proteins of PlasmoDB, HMMScan identifies 6 037 domains but leaves 2 068 proteins with no identified putative domains. CLADE drastically reduced this number to 1 544, providing 25% improvement and a global annotation of 7 841 domains (at E-values ≤ 1*e*-3; see Fig 5A and Table 2, top). These values describe the impact of CLADE on the full set of proteins. CLADE analysis on multi-domain proteins is even more impressive as reported in Fig 5A, where the importance of CCMs is highlighted. In many domain predictions, CLADE exploits CCMs in an exclusive manner: about 87% of CLADE domain predictions obtained with E-value ≤ 1*e*-60 are contributed by CCMs, and a total of 5 630 domains are identified by CCMs against 2211 identified by SCMs at E-value ≤ 1*e*-3 (Table 2, top). Also, more than a half of the domains predicted by CLADE are co-occurring domains and this is true for all predictions, independently on the E-values (Fig 5A). CLADE agrees on 98% of HMMScan predictions. Notice that HMMScan annotation is based on SCMs, and that if we consider CLADE SCMs predictions only, the number of CLADE predicted domains is smaller than that of HMMScan (2211 against 6037 domains). This is because CLADE predictions based on SCMs are often also obtained by CCMs with a better E-value and counted as CCM model predictions. The number of domains identified by SCMs and CCMs at a given threshold, is reported in Table 2 (top).

**Table 2:** Analysis of domains identified by CLADE but not by PlasmoDB.

	CLADE		SCM^d^		CCM^e^	
E-value	Cooc^f^	Total^g^	Cooc	Total	Cooc	Total
**All domains identified by CLADE**						
1e-60	843	1698	44	206	799/461	1492/801
1e-30	1858	3392	228	558	1630/1043	2834/1728
1e-15	3050	5329	527	1095	2523/1614	4234/2683
1e-5	4483	7172	1156	1823	3327/2230	5349/3555
1e-3	5167	7841	1544	2211	3623/2475	5630/3801
**Domains occurring on proteins predicted for the first time**^a^						
1e-60	7	23	0	0	7/4	23/18
1e-30	32	115	0	0	32/24	115/97
1e-15	127	393	2	2	125/110	391/361
1e-5	340	789	32	35	308/262	754/690
1e-3	467	916	57	60	410/346	856/774
**Domains enriching known protein architectures**^b^						
1e-60	41	49	0	0	41/37	49/43
1e-30	131	147	1	1	130/114	146/128
1e-15	347	428	2	2	345/294	426/373
1e-5	803	950	114	114	689/510	836/651
1e-3	1052	1200	206	206	846/638	994/780
**Brand-new domains in** ***P. falciparum*** **annotation**^c^						
1e-60	9	18	0	0	8/8	17/16
1e-30	51	103	1	1	50/44	102/91
1e-15	190	402	1	1	189/169	401/373
1e-5	448	816	40	40	408/353	776/712
1	603	971	79	79	524/458	892/817

For increasing values *E*, the number of domains is cumulative, all domains identified with an E-value ≤ *E* are counted.

^a^Number of CLADE predictions occurring on proteins with no domain annotation in PlasmoDB.

^b^Number of new domains annotated by CLADE that enrich already known protein architectures.

^c^Number of new domains annotated by CLADE that occur in no *P. falciparum* protein, according to PlasmoDB.

^d^CLADE new predictions obtained with sequence consensus models (SCMs). These domains are not identified by HMMScan with GA cut-off.

^e^Predictions obtained with clade-centered models (CCMs). Two values *n*/*m* are reported, *n* is the difference between the total number of CLADE predictions (third column) and the number of those predictions that are based on SCM (fifth column), and *m* is the number of predictions that are obtained exclusively by CCMs (within the E-value range).

^f^Number of CLADE predictions that are supported by domain co-occurrence.

^g^Total number of CLADE predictions including those that are not supported by domain co-occurrence.

**Fig 6:**
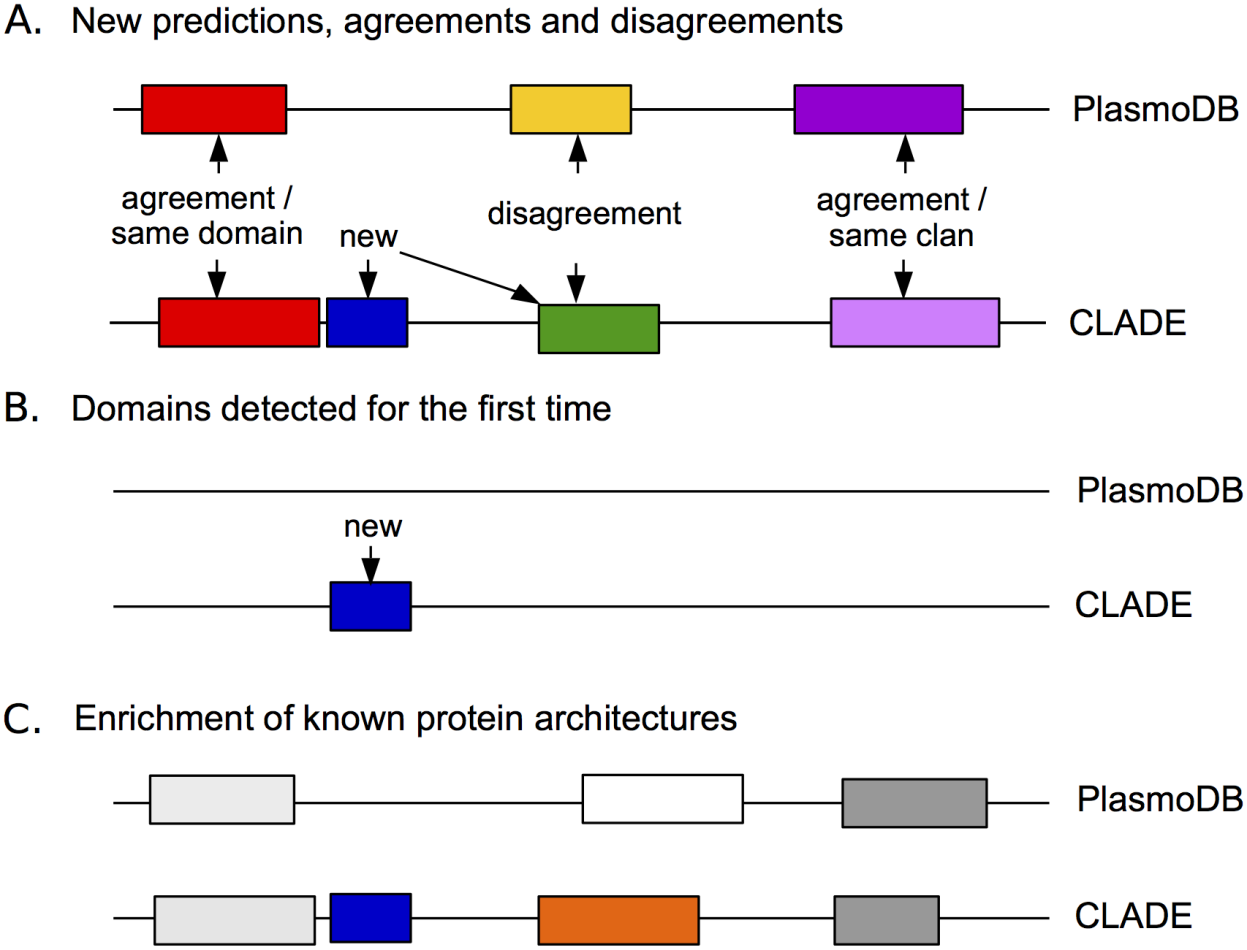
Schema of CLADE annotations versus PlasmoDB annotations. **A.** Examples of new domains in CLADE that overlap either non annotated regions (blue) or PlasmoDB hits associated to different clans (green and yellow). Red hits agree because they are associated to the same domain and (dark and light) violet hits agree because their associated domains are in the same clan. **B.** Example of a new domain in a sequence that is not annotated in PlasmoDB. **C.** Example of an enriched PlasmoDB architecture. It is constituted by CLADE domains that either agree with PlasmoDB domains (light and dark grey) or are new (blue and orange). Note that CLADE hits agreeing with PlasmoDB annotation might correspond to different regions in the sequence (see length of CLADE and HMMScan hits in dark and light grey).

Two overlapping hits in PlasmoDB and CLADE “agree” if they are associated to the same clan (possibly, the same domain). Vice versa, two overlapping hits “disagree” if they are associated to different clans (Fig 6A). We identify as “new”, those domains predicted by CLADE that do not overlap PlasmoDB hits (Fig 6B) or that disagree with some PlasmoDB annotation (Fig 6A). Also, we say that a CLADE hit is “brand-new” if the domain does not occur in any *P. falciparum* protein architecture in PlasmoDB. Based on these notions we observe that among the 2116 new domains identified by CLADE, 916 concern *P. falciparum* proteins that have never been predicted before (for an E-value ≤ 1*e*-3; Table 2) and 1200 are additional domains enriching already identified protein architectures (Table 2, middle). As expected, no contribution coming from SCMs seems to help, for both classes of predictions, most of the predictions being based on CCMs. 971 domains are identified as brand-new. Note that brand-new domains might enrich already known architectures or might occur in proteins with no annotation in PlasmoDB. CLADE predicts their co-occurrence with already annotated domains for 603 of them (Table 2, bottom).

Among the 2116 CLADE new domains (Fig 6A and 6B), there are 824 of them that belong to proteins with unknown function in PlasmoDB, and among the 1200 domains enriching known architectures (Fig 6A and 6C), we count 207 such domains (Fig 5B). In general, when considering predictions for proteins with unknown function in PlasmoDB, the majority of these predictions are realized without using domain co-occurrence and they greatly depend on the availability of CCMs (Fig 5B).

**Fig 7:**
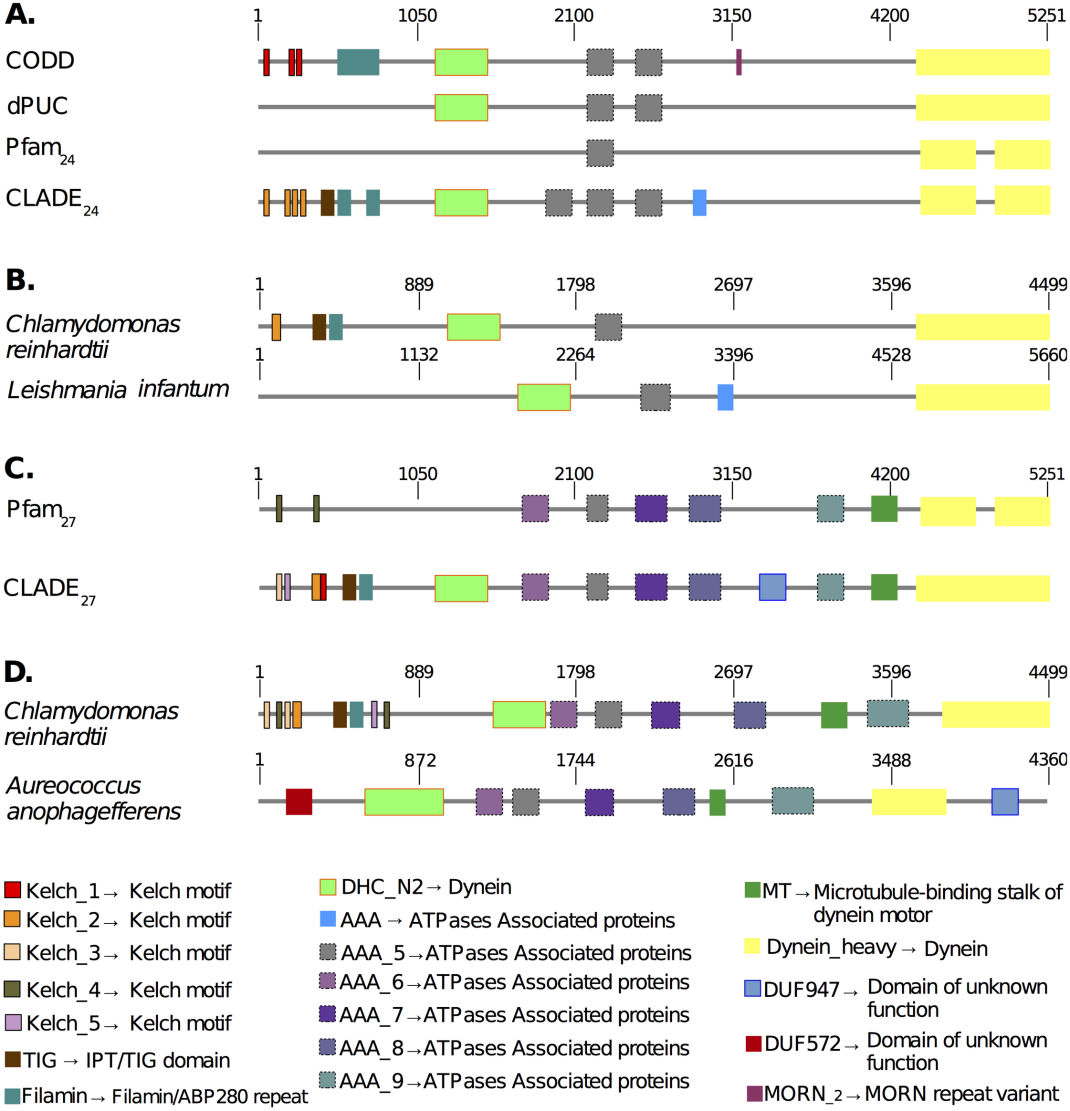
CLADE annotation supported by domain co-occurrence. Analysis of *P. falciparum* protein PF3D7_1122900 (PF11_0240). The protein is 5 251aa long. The list of domains (in various colors and) annotated by various methods is reported on the bottom. **A.** Comparison of the architectures found by CLADE_27_ (that is CLADE based on Pfam_27_ domains) and Pfam_27_. Six CLADE_27_ domains, absent in Pfam_27_, are highlighted by a black dot. **B.** Two Pfam_27_ architectures showing domain co-occurrence supporting CLADE_27_ annotation. **C.** Comparison of the architectures found by CLADE_24_ (that is CLADE based on Pfam_24_ domains), CODD [24], dPUC [26] and Pfam_24_. Notice that the MORN_2 domain has been predicted by CODD but that it is supported by an extremely high E-value (= 7.5) and no co-occurrence. All other domains have been detected by CLADE_24_ as well. Eleven CLADE_24_ domains, absent in Pfam_24_, are highlighted by a black dot. Some of these domains have been identified by CODD and dPUC also. **D.** Two Pfam_24_ architectures showing co-occurrence and supporting CLADE_24_ annotation. Compare with the *Chlamydomonas reinhardtii* annotation provided by Pfam_27_ in **B**.

An example of *P. falciparum* protein annotation realized with CLADE is illustrated in Fig 7. The sequence was annotated by Pfam_27_ with a few known domains. Besides confirming Pfam_27_ annotation, CLADE identifies new hits by co-occurrence of similar domains in other species (Fig 7A). The co-existence of Kelch motifs, TIG and filamin domains in a *Chlamydomonas reinhardtii* architecture and of DUF947 in a *Aureococcus anophagefferens* architecture with Dynein domain allowed for a more precise annotation than the one proposed by Pfam_27_ (Fig 7B).

This example bears witness to the coherence of CLADE predictions. In fact, it highlights that several domains identified by CLADE annotation based on Pfam_24_ and missed by Pfam_24_ annotation are confirmed by Pfam_27_ annotation. Namely, two of the four Kelch motif occurrences (orange domains in Fig 7C) and all three ATPases occurrences (grey domains in Fig 7C) identified by CLADE_24_ are confirmed by Pfam_27_ (olive green domains and violet, grey, dark violet domains in Fig 7A, respectively). Yet, a number of domains like DHC_N2 (Dynein), TIG and filamin, highlighted already by CLADE with CCMs generated in Pfam_24_, are still to be identified in Pfam_27_ annotation (Fig 7C). In this respect, notice that the *Chlamydomonas reinhardtii* architecture supported by Pfam_27_ has been highly enriched compared to the older version in Pfam_24_ (see Fig 7D). This demonstrates that domains co-existence is a very powerful tool of annotation analysis.

#### The multi-source strategy enriches CLADE architectures in *P. falciparum* sequences

**Fig 8:**
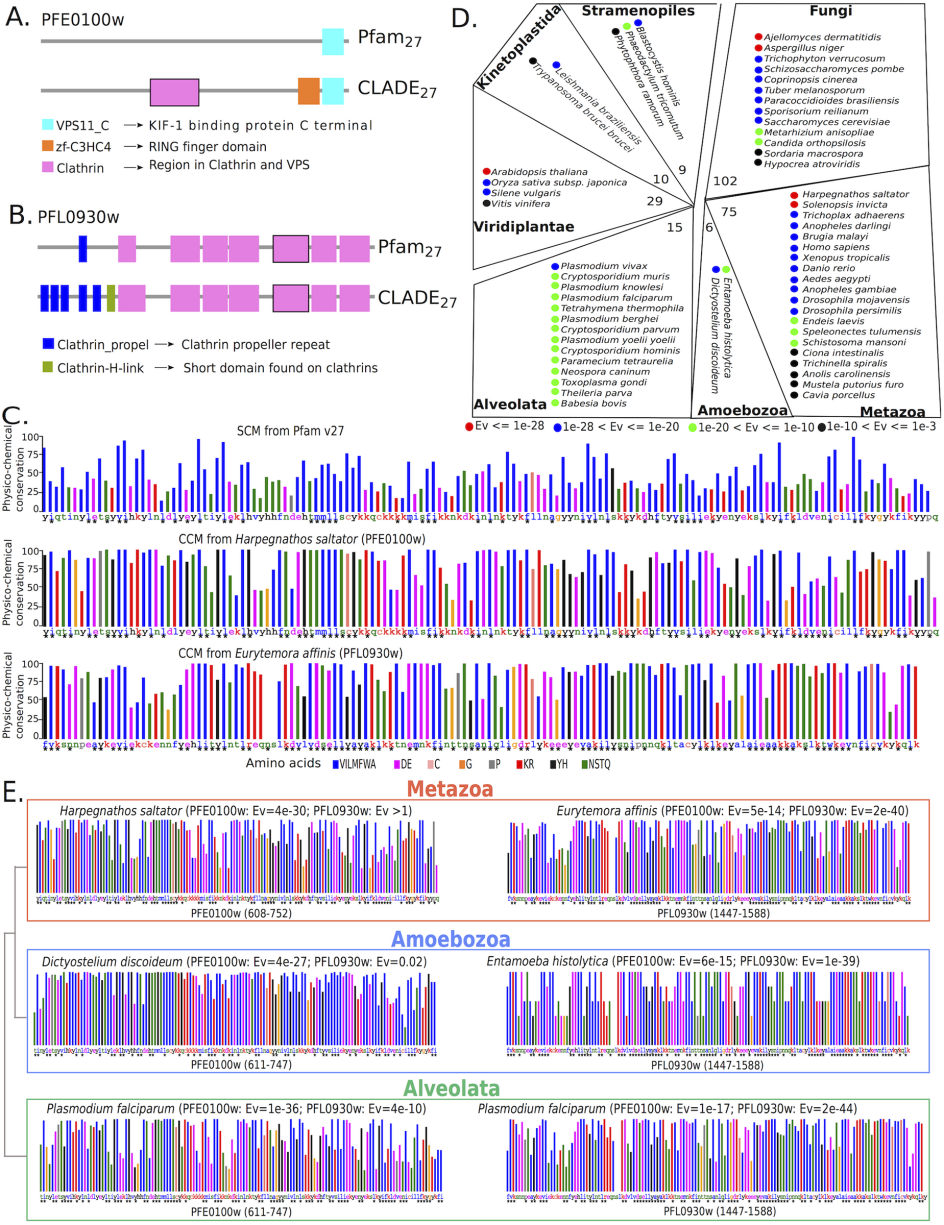
A concrete example illustrating the role of the multi-source strategy in CLADE. Analysis of the clathrin domain identification (Pfam domain PF00637) in two *P. falciparum* proteins PF3D7_0502000 (PFE0100w) and PF3D7_1219100 (PFL0930w). Their occurrence is highlighted with a black contour in **A** and **B**. These protein sequences are 1 272aa and 1 997aa in length, respectively. **A.** Annotation of PFE0100w. Pfam_27_ prediction: only one domain is identified. CLADE prediction: three domains are identified, two of which are new. **B.** Annotation of PFL0930w. Three domains are identified, a clathrin propel, a clathrin H-link and seven copies of the clathrin domain. Pfam_27_: the selected clathrin domain (with a black contour) is identified with E-value 1.9*e*-28. CLADE: the selected clathrin domain (with a black contour) is identified with a CCM based on *Eurytemora affinis* with E-value 2*e*-40. **C.** Physico-chemical conservation analyses of the SCM (top) for the clathrin domain identified in PFE0100w, and of the two CCMs generated by *Harpegnathos saltator* (middle) and by *Eurytemora affinis* (bottom) for the clathrin domain identified in PFE0100w and in PFL0930w, respectively. Each display shows the physico-chemical property that is most represented at a given position of the multiple sequence alignment generating the profile. Colored bars describe physico-chemical classes; the color scale is given at the bottom. The height of each colored bar corresponds to the frequency of the most represented (conserved) amino acid property. Values vary from 0 to 100: for instance, a blue bar that goes up to 100 indicates that the 100% of the amino acids occurring at this position are hydrophobic. The *P. falciparum* sequence PFE0100w is reported for the top and middle displays together with the physico-chemical positional matching, indicated by a *, between the corresponding residue in the *P. falciparum* sequence and the alignment position, if any. The display on the bottom is matched to the PFL0930w sequence. Amino acids are classified as suggested in [43] according to biochemical similarities: hydrophobic (VILMFWA), negatively charged (DE), positively charged (KR), aromatic (YH), polar (NSTQ), and C, G, P considered as special. **D.** The species that contributed to CLADE identification of the clathrin domain in *P. falciparum* sequence PFE0100w are organized into a schematic phylogenetic tree (drawing inspired by Figure 1 in [44]). For each clade, the total number of species involved in CCM construction is indicated at the “root” of the associated tree. Species are marked by a colored dot indicating the E-value range obtained by matching the corresponding CCM to the PFE0100w sequence. The color scale follows E-value ranges. **E.** Four amino-acid profiles of the clathrin domain, two provided by CCMs generated by Metazoan species (*Harpegnathos saltator* and *Eurytemora affinis*) and two by Amoebozoan species (*Dictyostelium discoideum* and *Entamoeba histolytica*), are mapped against the two *P. falciparum* sequences PFE0100w (left) and PFL0930w (right). (Note that PFE0100w and PFL0930w sequences are also reported in **C**.) E-values for the mappings of CCMs against the two *P. falciparum* sequences are reported on the profile heading (in parenthesis). Profiles in the Alveolata box (bottom) are constructed by running PSI-BLAST on the two identified *Plasmodium* domain sequences; they show high similarity with the profiles coming from Metazoan and Amoebozoan species.

To illustrate the power of the multi-source strategy, we discuss a concrete example of domain annotation for the two *P. falciparum* sequences PF3D7_0502000 (PFE0100w) and PF3D7_1219100 (PFL0930w), where a clathrin domain is found in the two sequences (see pink domains with a black boundary in Fig 8A and 8B) by two distinguished CCMs presenting rather different physico-chemical conservation profiles (Fig 8C, middle and bottom for domains in PFL0930w and PFE0100w, respectively). The SCM associated to the Clathrin domain by Pfam_27_ does not identify the domain in PFE0100w while it identifies it in PFL0930w with E-value 1.9*e*-28.

We observe a low level of conservation in the physico-chemical profile of the Pfam_27_ SCM (Fig 8C, top) compared to the high level of conservation recorded for the two CCMs used to annotate the two sequences (Fig 8C, middle and bottom): when aligned with the clathrin hit in the respective *P. falciparum* sequences, one observes that the number of positions preserving the physico-chemical properties in *P. falciparum* is much higher for the CCMs than for the SCM (see the higher number of starred positions below each one of the three profiles).

Besides the CCM used to best annotate the PFE0100w sequence, all other CLADE CCMs for the clathrin domain were considered against the PFE0100w sequence but they scored lower than the CCM generated by *Harpegnathos saltator*. The distribution of their E-value scores across the phylogenetic tree is illustrated in Fig 8D. We notice that the best hits were obtained with CCMs generated by Metazoan or Fungi species, and that for all models generated by Alveolata species, scores were quite low, suggesting that annotation based on Alveolata species could be hard to identify. A similar pattern can be displayed for PFL0930w.

Several CCMs display a physico-chemical conservation profile that is closer to PFE0100w than to PFL0930w. See Fig 8E. For both sequences, we find CCMs with close profiles in the three Metazoa, Amaebozoa and Alveolata clades.

#### Species helping domain identification

To understand how the large space of available sequences is exploited by CLADE to attaint *P. falciparum* predictions, we performed two distinguished *a posteriori* analyses of the set of species generating the CCMs that helped CLADE predictions (Fig 5C).

First, we observed that the 54% of the contribution is provided by homologs belonging to the Alveolata clade and that the 46% of homologs belongs to other clades and, among them, Metazoa, Fungi, Viridiplantae appear to be the most represented Eukaryotic clades. A non negligible contribution is also recorded from Viruses, Bacteria and Archaea homologs. (Compare Bacteria with Viridiplantae in the inset of Fig 5C.) This finding agrees with the intuition that best sequence similarity most likely appears within the Alveolata clade, but it also highlights that half of the times this is not the case, and especially for the predictions whose E-value is not very high (notice the E-value distribution in Fig 5C). When we consider new domains only, the contribution from Alveolata species decreases (32.21%) in favour of all other species (67.79%; S4 Fig). Over 2 116 new predictions, 291 come from CCMs generated from viral, bacterial and archaeal species.

In this respect, the analysis reported in Fig 4C quantifies the improvement obtained by adding to the Pfam set of models, those CCMs generated by sequences belonging to arbitrary species (red curve in Fig 4C) compared to the Alveolata models only (grey curve Fig 4C), making the 3% of all CLADE models (see Fig 2). It confirms the interest in an expanded model construction.

Second, we checked whether pairs of domains occurring in the same *P. falciparum* protein were identified based on CCMs associated to species belonging to the same clade or not (S3 Table). Over the 1 688 *P. falciparum* proteins with at least two domains identified by CLADE (where the confidence on these predictions is strongly supported by domain co-occurrence), we found that 900 multi-domain proteins contain domains that are detected by CCMs coming from different clades. In particular, if we look at predictions with respect to a fixed E-value, we find that among the 60 multi-domain proteins containing at least a domain predicted at E-value ≤ 1*e*-60, 28 present a domain identification that is based on different clades. Similarly, among the 627 multi-domain proteins whose domains are identified with 1*e*-15 < E-value ≤ 1*e*-5, 376 are identified with different clades, that is almost half of the proteins containing at least two domains are identified with the help of several clades. This is in agreement with the hypothesis that a protein evolutionary process is random, and hence, clade independent. A concrete example is illustrated in Fig 8, where CLADE identifies three domains with CCMs generated by sequences from Metazoa (*Harpegnathos saltator* for the clathrin domain), Trichomonadida (*Trichomonas vaginalis* for zf-C3HC4) and Alveolata species (another *P. falciparum* protein for VPS11_C). Two of the species defining CCMs predicted with best E-values are sparse in the tree and far from the Alveolata clade, explaining the difficulty of annotating this protein with SCMs. These three domains have been found to co-occur in 34 different species including *Homo sapiens* and *Anopheles gambiae* (details are available in Pfam website).

#### Physico-chemical conservation for predictions based on CCMs

CCMs used in domain prediction provide a physico-chemical profile of the protein that is typically more conserved than the one obtained by the associated SCM (observe the number of starred positions along the *P. falciparum* sequence in Fig 8C).

The amino acid groups analysis of the two *P. falciparum* sequences, PFE0100w and PFL0930w, shows very high positional conservation obtained with the CCMs of *Harpegnathos saltator* (E-value 4*e*-30) and *Eurytemora affinis* (E-value 2*e*-40). Compare with the conservation profile of the SCM on the PFE0100w sequence (E-value > 1, top).

This property augments our confidence in the method. In particular, notice that physico-chemical profiles help to detail the differences between CCMs. In fact, different matches of different CCMs for the same domain might highlight different evolutionary signatures of the domain. In Fig 8E, for instance, the physico-chemical profiles of different models for the clathrin domain indicate two classes of models, one matching the PFE0100w domain and the other matching the PFL0930w domain (see left and right profiles in Fig 8E).

#### CLADE versus HHblits on *P. falciparum* genome

We compared the domain annotation obtained with HHblits and CLADE. For this, we considered increasing HHblits FDR thresholds (from 0.1% up to 10%) and fixed CLADE FDR at 0.1%. Looking at predictions upon which CLADE and HHblits agree (overlapping hits that are annotated by the two systems with the same domain or by domains belonging to the same clan; see S5 Table), we observe that:

1. the proportion of HHblits domain hits in agreement with CLADE is much larger for stringent FDR thresholds (note the almost complete overlapping at FDR 0.1% reported in S5 Table) and gets smaller, by reaching a converging bound, for high FDR values.
2. CLADE can detect with higher confidence (due to smaller E-values and co-occurrence), domains that are detected by HHblits at much higher FDR, and therefore much lower confidence. For instance, of the 916 CLADE domains occurring on proteins annotated for the first time, 477 of them are detected by HHblits at FDR 10%.
3. CLADE predicts in newly annotated architectures more than 400 domains that are missed by HHblits at FDR 10%. Of those, 230 are supported by co-occurrence and have E-value varying from 6*e*-88 to 1*e*-3. The fact that they are supported by known co-occurrent domain contexts brings confidence in their prediction.
4. for high FDRs, HHblits is prone to obtain a large number of false positives. In fact, note that at FDR 10%, only 772 domains over a total of 3 260 HHblits predictions on proteins annotated for the first time, are supported by domain co-occurrence. This holds true for both the enrichment of known protein structures and the predictions of brand-new domains. In contrast, notice that at a FDR of 0.1%, many HHblits predictions are supported by co-occurrence.

## Discussion

When sequences in a protein domain family are too divergent, signals of homology are easier to trace with CCMs rather than with models based on the consensus of homologous sequences spanning the entire phylogenetic tree (reminiscent ideas were presented in [7, 18–20, 45]). CLADE shows that, by combining in a unique tool CCMs and consensus models, the predictive power can be highly reinforced.

In CLADE, CCMs have been constructed for domains in the Pfam database. In this respect, it is worth to highlight that CCMs are basically different from: 1. Pfam models characterizing Pfam clans [46] whose purpose is to group different families together, possibly new protein families that appear to have arisen from a single evolutionary origin; 2. Pfam models characterizing Pfam domain families (called SCMs in CLADE). Compared to both Pfam family models, CCMs are much closer to actual protein sequences, therefore more specific and more functionally predictive. In fact, one can think of CCMs as forming a new layer of models situated at the very bottom of the Pfam hierarchy and associated to FULL sequences within Pfam domain families. The example reported in Fig 8C and 8E illustrates how CCMs are closer to sequences than Pfam family models.

Standard approaches, like Pfam, are limited to consensus within seeds only, and CCMs showed to model evolutionary information differently. Below, we propose several ways to revisit basic evolutionary questions based on CCMs.

**CCMs can be used to analyze ancient duplications.** Modern sequences evolved in the context of full genome and local duplications. The fate of the duplicated domains could be different for several reasons. The most important one is domain organization within architectures, where the accomplishment of different cooperative functional purposes might induce duplicated domains to reach possibly very divergent sequence profiles. CCM can help to model these parallel profiles (created by domain divergence) better than a consensus approach. The example on clathrin domains illustrated in Fig 8 highlights the parallel co-existence in the Metazoa and Amoebozoa clades of CCMs characterized by two distinguished profiles, suggesting that an ancient duplication took place before the Metazoa-Amoebozoa phylogenetic divergence and gave origin to the two kinds of domains observed today in the two clades. The existence of two distinguished copies (fitting the two profiles) of the clathrin domain in the *P. falciparum* genome, reveals that this duplication is even older and places it before the Metazoa-Amoebozoa-Alveolata divergence. CCMs can be used for large scale explorations of parallel profiles evolution.

**Multiple CCMs can be used to analyse domain evolution.** Whether the actual number of evolutionary pathways for a domain family is relatively small or not remains an open question. The large number of CCMs associated to a domain *D* characterizes the evolutionary landscape of *D*, and highlights the viability of different evolutionary processes. The mathematical description of the models, as probabilistic profiles, can be used to explicitly address quantitative questions on the landscape variability. For instance, a measure of how different CCMs are, one from the other, can be developed and used to bring an estimation on the number of distinct evolutionary pathways associated to a domain. Do different domains have a sensibly different number of evolutionary pathways associated to them? Does the distribution of distances between probabilistic profiles of a given domain have a small/large variance? Can we suggest a graph-like relational structure among CCMs and exploit the structure of the graph to infer functional consequences? These questions do not have an answer today but they seem fundamental to the understanding of the evolutionary landscapes we observe.

**CCMs and their impact on functional annotation.** The interest of an accurate genomic mapping of protein domains and protein architectures is multiple. It directly implies the possibility to develop: 1. a more precise functional analysis of domains and architectures within genomes; 2. a comparative analysis of domains and architectures between species within clades. Based on it, pangenomic differences of phylogenetically close microbial species (strains or genomes) can be defined at the domain level, and species variability carefully assessed. By using domains as the building blocks of proteins functional activity, we can assert that the presence of the same domains within different architectures in different species/strains might guarantee a similar functional activity for the organism. In this sense, a relaxed notion of pangenomic variability can be defined, closer in spirit to the functional activity of the species. Similar observations apply to microbial communities, where domains, more than proteins, appear to be useful building blocks for functional annotation; 3. a comparative analysis of domains and architectures between species across distant clades. This could help to improve estimating the age of domains and architectures [47, 48]; 4. an accurate identification of gene homology between pairs of genomes. This will directly benefit synteny blocks reconstruction and chromosomal rearrangement analyses; 5. an improved tracing of gene acquisition for bacterial species, where lateral gene transfer is much present. This will imply a more precise reconstruction of reticulate evolution.

**CCMs and domain architectures identification.** The way protein architectures form is an important factor to understand protein evolution. A quantification of the elementary events affecting protein architectures, such as domain(s) insertion/deletion, duplication and exchange, was done [49] but, yet, little is known about the relationships between these elementary events [50] and the molecular mechanisms they originate from. Finer domain mapping (obtained with more precise annotation tools as CLADE) on all proteins of completely sequenced genomes will contribute precise information on the evolution of protein architectures. This means, for instance, a more precise estimation of the rate of insertion, deletion, duplication and exchange of domains within proteins in a given species. In general, it would be interesting: 1. to establish whether the process of generation of an architecture follows constraints or not, 2. to pinpoint such constraints, if they exist, and 3. to verify whether they are species specific or not. This information turns out to be useful in the context of phylogenetic profiles prediction [51].

**The role of the Alveolata and other clades in *P. falciparum* domain annotation.** An *a posteriori* analysis of our predictions highlights that: 1. species in the Alveolata clade are preferably chosen for domain identification (54%), 2. a significant number of identifications (46%) are suggested by species that lie outside the Alveolata clade (that is, far from *P. falciparum*) and yet providing acceptable E-values for predicted domains (Fig 5C). The first point confirms the strength and importance of considering phylogenetic signals in annotation, and the second point highlights the limitations of the idea of phylogenetic proximity.

Besides the observation that many *P. falciparum* sequences are more easily identifiable by CCMs generated by phylogenetically distant species rather than CCMs coming from Alveolata species, other observations strongly reinforce the interest in looking at different clades while identifying domains: 1. multi-domain proteins identified with CCMs originated in different clades, suggest processes of domain evolution that are independent within each protein (see the three domains identified with models constructed from Alveolata, Amaebozoa and Cryptophyta sequences in Fig 8); 2. new domain families are periodically added to databases like Pfam and annotation becomes gradually more precise as a function of this addition (see the impact of the Pfam_27_ enrichment on the *Chlamydomonas reinhardtii* protein architecture compared to Pfam_24_ in Fig 7A and 7C); 3. CCMs coming from bacterial and archaeal species should allow to check for ancient lateral gene transfer.

**On CLADE methodological improvements.** On the methodological side, the use of agreement among models (handled by SVMs) and of co-occurrence (handled by DAMA) in CLADE improves predictions up to 19.6% on the *P. falciparum* sequences (S4 Table), over a score system based on best E-values (CLADE_*BEv*_) and this highlights that criteria other than sequence similarity, play a key role in the identification of protein domains. The usage of multiple criteria to reach agreement among models, allows for new predictions and plays an important role in the evaluation of the confidence in a prediction, with improved scores that can help the biologist to annotate sequences.

Several ways could be envisaged to improve further our methodological approach.

1. The sequence search tool PSI-BLAST [52] could be replaced by the profile-profile comparison tool HHblits [17], and both CCMs and consensus models could be constructed by HHblits. For large applications, the construction of profiles for the query sequences and the identification of domain specific cut-offs for all domains become too costly, and one could transform HHblits models in HMMs to avoid profile-profile comparison.
2. The number of meta-features in the meta-classifier used to combine model predictions could be increased. For instance, one could encode motif information known for specific domains. The SVM could be improved, by making the choice of a given kernel to be domain specific. More radically, the choice of designing CLADE based on SVM could be revisited. Other decision strategies could be employed instead, like multi-response linear regression [53] for instance.
3. Domain co-occurrence re-ranks low confidence domains as relevant when they appear with co-occurring domains. The power of this criterion has been already observed in [24, 26, 34, 35, 54, 55]. Other multi-objective optimization functions could be added in DAMA to screen the domain list and exploited in CLADE, as discussed in [27].
4. CLADE selects a reference set of domain sequences exploiting the large span of organisms in the phylogenetic tree of life. This choice ignores how protein evolution is full of gene duplications, horizontal gene transfer, domain shuffling and so on. It is reasonable to think that building gene trees and couple them with CLADE would help to better span the sequence diversity right at the beginning of the models construction. Future development of CLADE could explicitly consider orthology/paralogy/xenology to better build CCMs.
5. The pool of approximately 350 CCMs associated to each domain could be extended to provide an enriched annotation. Several databases, such as SCOP, CATH, Gene3D, propose domains that are not shared with Pfam. From these domains new models can be constructed and added to the CLADE model library. This perspective demands the development of criteria to avoid redundancy in the model library and to ensure a reasonable computational time.

These methodological improvements are general, independent from specific genome characteristics, and guarantee the strategy to be applied to any genome. They will likely be able to address at least a part of the 28% of *P. falciparum* proteins that are still missing a domain identification and the protein architectures that should be enriched with new domains (among the 2394 single domain proteins, 1214 of them contain a domain that covers less than the half of the protein length, and for these proteins we expect multiple domains to lie together). Some unannotated proteins in *P. falciparum* likely contain completely novel domains, which are evolutionarily unrelated to domains present in the existing domain family databases. If that is so, CLADE would not find them, at least not until existing databases grow enough to cover more of that domain space.

**CCMs and computational power.** By using HPC, CLADE demonstrates to push the limits in annotation reached by current methods. The first step of CLADE, generating CCMs, is the highly expensive one. It is performed once for all genomes to be annotated. All CCMs used to realize *P. falciparum* annotation have been constructed in 3.7 months of computer time by using 250 CPUs (and they are made publicly available). The two subsequent steps (2 and 3), dedicated to genome annotation, are relatively fast. For example, CLADE steps 2 and 3 ran in about 1 hr on 100 CPUs for the entire *P. falciparum* genome. CLADE domain library is expected to be regularly updated on new domains appearing in databases. This means that only CCMs for new domains need to be constructed and added to the existing library. This step can be realized independently from steps 2 and 3. In years to come, the expected improvements in HPC and in CLADE implementation (with a thoughtful selection of domain models) will render CLADE more computationally accessible.

## Materials and Methods

### Databases

Our method extends Pfam, an important collection of protein domains, that has been widely used for annotating proteins with unknown function. We use Pfam v27 (Pfam_27_, downloaded from http://pfam.sanger.ac.uk), containing 14 831 protein domains. In order to assess the performance of our method, we apply it to the set of all *P. falciparum* proteins. For this, we use PlasmoDB (http://PlasmoDB.org), that is the official repository of the *P. falciparum* proteins used as a reference database by malaria researchers. PlasmoDB v11.1 contains 5 542 proteins.

We used the UniProtKB database [56]: 1. to extract NCBI taxonomy for sequences and the list of known domain architectures. 2. to recover the Pfam domain organization of all proteins in UniProt 15.6 (Swiss-Prot 57.6 and SP-TrEMBL 40.6); we downloaded the dataset ftp://ftp.ebi.ac.uk/pub/databases/Pfam/releases/Pfam27.0/swisspfam.gz and used it to analyze co-occurrence in CLADE annotations. (In our tables and figures, “Cooc”, abbreviating “predictions supported by domain co-occurrence”, and “Cooc-CCM”, abbreviating “predictions identified by a CCM and supported by domain co-occurrence”, count architectures whose domain pairs belong to already known architectures.)

A reference list of clades has been extracted from NCBI (http://www.ncbi.nlm.nih.gov/taxonomy) and used for selecting a representative set of sequences in the construction of CCMs. Clades have been specified for Bacteria, Archaea, Viruses and Eukaryotes in S1 Table.

We used the SCOP v1.75 [41] database to compare CLADE, based on the multi-source strategy, with the mono-source strategy of HMMScan [2, 33] and HHblits [17]. Also, the SCOP datasets allowed us to compare CLADE with the computational strategy employed in SUPERFAMILY, a system that builds multiple hidden Markov models, for each protein superfamily, to realize sequence search. The SUPERFAMILY sequence search method is built on 1 962 superfamilies (from classes a to g), while CLADE relies on Pfam_27_ containing 14 831 protein domains. To realize the comparative analyses, we considered SCOP domains whose associated sequences are coming from at least 10 species, and constructed a three testing sets from the ASTRAL95 dataset, containing 255 domain families and 8 633 sequences with at most 95% sequence identity, from ASTRAL30, made of 66 domain families and 1 251 sequences with at most 30% sequence identity, and from ASTRAL10 made of 18 domain families and 306 sequences with at most 10% sequence identity. ASTRAL95, ASTRAL30 and ASTRAL10 are subsets of SCOP [21] and can be downloaded from http://scop.berkeley.edu/astral/subsets/ver=1.75.

Tests with HHblits required the use of HHdb, a database built from the UniProt and NCBI NR databases, and provided in the HH-suite package [17, 42].

### Tools run for comparison

HMMScan was run with default parameters and curated inclusion thresholds. The option –cut_ga, for model-specific thresholding (using profile’s GA gathering cutoffs to set all thresholding), was used. HMMScan is included in the HMMer 3.0 package [33] downloadable at http://hmmer.janelia.org/software.

HHblits was run with default parameters. It is part of the HH-suite package—version 2.0.15 [17, 42] downloadable at https://github.com/soedinglab/hh-suite.

### Detailed description of the CLADE pipeline

First, we detail how Pfam methodology works and how we modify it by including additional models, called *clade-centered models* (CCM). Then, we describe how to combine those models to produce reliable predictions. In the final step, we apply DAMA, an algorithm especially designed to take into account domain co-occurrence, to find the most likely domain architecture.

#### Pfam methodology

Let *D*^*i*^ be an arbitrary protein domain in the Pfam database. In Pfam, *D*^*i*^ is associated to two sets of sequences, SEED and FULL. SEED is a set of protein sequences, called seed sequences for the domain *D*^*i*^, that share evolutionary and structural properties. It is defined by Pfam curators and results in a high-quality set of sequences. Pfam aligns seed sequences to build a profile hidden Markov model, SCM^*i*^, that represents the consensus of the seed alignment, that is the common features of the seed sequences. SCM^*i*^ is used by Pfam to scan databases of proteins with unknown function by localizing regions in sequences that are similar to the domain *D*^*i*^. Pfam predictions are those domain hits that HMMScan identifies with the –cut_ga option. In contrast, FULL is a automatically generated set. To construct it, Pfam uses SCM^*i*^ to search in UniProtKB [56] for all significant hits from HMMScan (using the –cut_ga option). Most FULL sequences are never evaluated by experts, and some of them are probably false positives from HMMScan (sharing neither evolution nor structure). FULL contains SEED. In what follows, FULL is denoted $S^{i}=\left\{s_{1}^{i},\ldots ,s_{n}^{i}\right\}$ and SEED is denoted $S^{i*}=\left\{s_{1}^{i*},\ldots ,s_{m}^{i*}\right\}$. Each sequence in FULL, and therefore in SEED, is associated to a protein in the UniProtKB database and provides a NCBI taxonomy for it.

A simplified Pfam flowchart, showing the library of SCMs generated by HMMer, one for each domain *D*^*i*^, is illustrated in S1 Fig (solid lines).

#### Clade-centered models

SCM^*i*^ is a probabilistic model explaining how sequences in the SEED dataset *S*^i^* of Pfam_27_, associated to the domain *D*^*i*^, have evolved. By contrast, we shall build a number of new models by exploiting information coming from (possibly different) evolutionary paths associated to specific species. They are called $\text{CCM}{}_{j}^{i}$, where CCM stands for *clade-centered model*, *i* indexes the domain *D*^*i*^, and *j* is an index running over the different models. To construct them, we take at most 350 sequences belonging to different species by choosing them from the FULL dataset *S*^*i*^ in Pfam_27_. The algorithm extracts a comparable number of sequences, whenever possible, from each clade. This automatic selection guarantees that the distribution of species is as uniform as possible across the panel of phylogenetic clades. (See list of clades leading the selection in S1 Table.) This set of selected sequences is referred to as $\bar{S^{i}}$. Each sequence in $\bar{S^{i}}$ will be used as a query to search for similar sequences, with PSI-BLAST [52], within the non-redundant protein database (NR). From the resulting set of similar sequences, a probabilistic model $\text{CCM}{}_{j}^{i}$ is constructed. As a result, we produce models $\text{CCM}{}_{1}^{i},\ldots ,\text{CCM}{}_{m_{i}}^{i}$, with $m_{i}=\left|\bar{S_{j}^{i}}\right|\leq 350$. See S1 Fig (dotted lines).

#### Combining Model Predictions

Reliable predictions are obtained by combining domain hits from different models. We follow three main steps:

*1. The building phase of the ensemble of clade-centered models.* We modify Pfam original library, in such a way that, a single domain *D*^*i*^ is now represented by an *ensemble of models* $C^{i}=\{\text{CCM}{}_{1}^{i},\ldots ,\text{CCM}{}_{m_{i}}^{i},{\text{SCM}}^{i}\}$. We have used PSI-BLAST [52] and HMMer [33] for providing models in $C^{i}$. The two tools exploit different data: individual sequences in the FULL dataset of Pfam_27_ were used as queries to build $\text{CCM}{}_{j}^{i}$ with PSI-BLAST, and sequences in the SEED dataset were used by HMMer in Pfam_27_ to build SCM^*i*^. SCM^*i*^ models were downloaded directly from the Pfam website. A flowchart describing this pipeline is illustrated in S1 Fig (dotted and solid lines).

*2. The meta-classifier training phase.* After the building phase of CCMs and SCMs, the models are run to search for potential domains and model outputs are combined to produce a final decision. For this latter step, one can employ plurality voting or meta-learning techniques [57]. We implemented a meta-learning decision strategy that used a meta-classifier (Support Vector Machine—SVM) trained from features that were obtained by pre-processing outputs of CCMs and SCMs. Model outputs (frequently corresponding to confidence scores) have been used directly in the SVM training [58]. After trying this algorithmic approach, we realized that we did not achieve good performance possibly for two reasons. First, the high divergence of the sequences in a protein dataset (for instance, the *P. falciparum* protein dataset) can imply that the distribution of confidence scores in the training set is different from the distribution in the testing data. Second, our models are very heterogeneous, since they were trained from different data sources, thus, it is not expected that there is necessarily an agreement among their predictions. Because of these two observations, any decision strategy that tries to find a perfect agreement of model answers is expected to fail. To avoid this, we designed meta-features which aim: (i) to highlight individual model results, when agreement among CCMs and SCMs models is not observed, and (ii) to provide an indication of the performance of all models. See schema in S2 Fig. We defined five meta-features as follows.

Let *s* be a query sequence that we wish to score against all models in $C^{i}$. Since the domain *D*^*i*^ can be found in multiple copies within *s* by different models $C_{j}\in C^{i}$, where *C*_*j*_ is one of the CCMs or SCM^*i*^ in $C^{i}$, we want to identify all best matches in *s* that do not pairwise overlap. For each one of these best matches we create a distinguished hit, described by the location on the sequence and by 5 features of the associated model *C*_*j*_. To achieve goal (i), we extract three features from the *C*_*j*_ output: the E-value (provided either by HMMer for SCMs, or by PSI-BLAST for CCMs), the hit length (that is, the length of the domain found in *s* by the model *C*_*j*_), and a binary feature that indicates if the E-value of *C*_*j*_ is smaller than a threshold *T*′. For goal (ii), we define two features concerning the percentage of models in $C^{i}$ that *support* the prediction of *C*_*j*_. For this, we say that a model *C*_*l*_ supports the prediction of a model *C*_*j*_ if their matches on *s* overlap each other and the overlapping size is greater than 50% of the *C*_*j*_ match size. Thus, the fourth meta-feature is defined as the percentage of models that support *C*_*j*_ having E-values smaller than a threshold *T*′′. The fifth feature represents the percentage of models *C*_*l*_ that support *C*_*j*_ and that are built from species that belong to the clade of *s*. Our motivation is based on the assumption that species of the same clade tend to share more domains than species of different clades. Note that, we do not penalize predictions that are obtained from models built from species of distant clades, but we just wish to use this evolutionary information to reinforce the presence of a domain when it is observed in species close to *s*. We discuss *T*′ and *T*′′ thresholds in section “CLADE pipeline, parameter settings and tools used in CLADE” (Methods).

For each domain *D*^*i*^, we trained a meta-classifier (SVM) from the five meta-features described above, to distinguish between real domain occurrences and false predictions. More precisely, we built a one-vs-rest SVM^*i*^ [59], that is a binary classifier that discriminates two classes by finding a large-margin separation among them, as illustrated in S2 Fig. For this, we used all sequences in the set $S^{i}\setminus \bar{S_{j}^{i}}$ as a positive training set, that is all sequences in *S*^*i*^ with the exception of those used to build CCMs. If $|S^{i}\setminus \bar{S_{j}^{i}}|<50$ sequences, then we consider the entire set *S*^*i*^. Moreover, to augment the number of sequences in the positive training set, we apply a rate of mutation of 20% on the initial set of sequences $S^{i}\setminus \bar{S_{j}^{i}}$ (*S*^*i*^ when $|S^{i}\setminus \bar{S_{j}^{i}}|<50$) to arrive to a total of 1 000 sequences per domain. This was done with The Sequence Manipulation Suite software [60]. To construct the negative training set, we randomly choose 20 sequences in each set *S*^*k*^, where *k* = 1…14 831 (the number of Pfam domains), and shuffle their 2-mers. We obtain about 300 000 sequences. Notice that when we compare models of *D*^*i*^ against these 300 000 sequences, only a few of them give an E-value smaller than 1 (chosen as threshold) and this ensures these sequences to be negative for *D*^*i*^. We repeat the construction 10 times and randomly choose, among all generated sequences, 1 000 sequences that will form the negative set. Positive and negative datasets have the same size, in order to avoid unbalanced sets. (For certain protein families this is obtained by using the supersampling SVM option.)

*3. The prediction phase.* Each protein sequence in the set to be annotated is used as a query *s* to be scored against all models in the ensemble $C^{i}$. The hits produced by the models (corresponding to predictions of domain *D*^*i*^ in the sequence *s*) are then processed and the five features described above are extracted for each occurrence of domain *D*^*i*^ in *s*, as indicated in S2 Fig. Then, the SVM^*i*^ (trained to recognize the domain *D*^*i*^, as described above) is asked to determine if the domain *D*^*i*^ is found in the protein sequence *s*, and to provide a confidence score. However, from a biologist’s perspective, it is more valuable to identify the most likely domains that occur in *s* and that are not overlapping. This is known as a multi-class classification problem. To enable a set of one-vs-rest SVMs (one for each domain) to work with this problem, it is essential to calibrate the output of each classifier into a confidence measure, like the posterior probability. Since standard SVMs do not provide such probabilities, we employed Platt’s method [61] to map SVM outputs into posterior probabilities. As a result, SVM’s probabilities are comparable to each other, and we can assign to *s* the domain that achieves the highest predictive value, as done in [62]. Strictly speaking, as noticed above, if several non-overlapping matching of the same domain *D*^*i*^ are found in *s*, then for each non-overlapping match, we assign to *s* the domain that achieves the highest predictive value.

At the end of this step, several overlapping hits associated to different potential domains are identified for *s*. Based on this set of potential domain hits, we determine the most likely domain architecture for *s*. For this, we first observe that the tendency of a domain to occur preferentially with a small set of other domains in a protein sequence can favor lower confidence domains compared to higher confidence domains, once we want to insert them in a domain architecture. Hence, we applied DAMA, an algorithm that has been designed to determine the most probable domain architecture for *s* by taking into account domain combinations. Its performance has been evaluated in [27].

#### E-values estimation in CLADE

CLADE works with two kinds of probabilistic models, generated by PSI-BLAST and by HMMer. Because of their format we cannot put them together in a single library and handle them in the same manner. A possibility would be to convert PSI-BLAST to HMMer format or vice-versa. However, the transformation could decrease the performance of the models. Then, we score the models individually, that is, each model is used to scan the entire set of query sequences given in input to CLADE and the E-values corresponding to the matches are estimated by the respective tools (PSI-BLAST and HMMer). This strategy assumes models to be independent. It should be noticed that this independence assumption is frequently adopted by machine learning methods and it has been shown to work well in most cases [63]. By adopting this strategy we assure CLADE E-values not be over-estimated.

#### CLADE pipeline, parameter settings and tools used in CLADE

The three steps constituting CLADE are implemented with a number of parameters and implementation choices:

1. For each Pfam domain, an ensemble model, containing several CCMs and a SCM, is constructed and used for domain identification; an E-value is provided for each identified domain.
2. Identified domains are filtered, based on several criteria; SVM probabilities are computed for each domain.
3. Best architectures are selected using DAMA, and domains appearing in an architecture are identified by an E-value and a SVM probability.

SCMs were downloaded directly from the Pfam web site, while CCMs were built with PSI-BLAST (v.2.2.28 for 5 iterations and E-value cut-off 1*e*-03) on the NR database (download in March 2014). PSI-BLAST was applied to sequences coming from species that were representatives of the whole tree of life. In its second step, CLADE searches for domains occurrences in the query sequences, based on CCMs and SCMs. In order to detect a large number of potential Pfam domains, we set a permissive search E-value (= 1) in PSI-BLAST and HMMer search, using default values for the remaining parameters. We combined the predictions obtained by all profiles by training a SVM from features coming from profile outputs. For SVM, we used the LIBSVM tool [64] (v.3.0) with default parameters, and we turned on the option “-b” to provide probability estimates. The software is available at http://www.csie.ntu.edu.tw/~cjlin/libsvm.

Five features were designed to highlight the best prediction and to provide a measure of the performance of all profiles (see section “Combining Model Predictions” in Methods). The definition of two of these features depends on the thresholds *T*′ and *T*′′. Namely, the restrictive E-value threshold *T*′ = *min*(1*e* − 30, *Ev*_*D*^*i*^_), where *Ev*_*D*^*i*^_ is the greatest E-value observed among proteins containing the domain *D*^*i*^ in Pfam sequences, is set to compute the third feature. The fourth feature equals the number of hits (profile outputs) with E-value smaller than *T*′′ = 1. This is a permissive threshold used to count the agreement with best predictions.

For each domain *D*_*i*_, a specific SVM probability cut-off *T*_*D*_*i*__ was defined, as done in Pfam [2]. Namely, we considered all sequences in the positive training set for the domain *D*_*i*_ defined at the end of subsection “The meta-classifier training phase” (see section “Combining Model Predictions” in Methods) and computed SVM probabilities for each sequence in both the positive training set and the negative training set. If the intervals of the positive and the negative distributions do not overlap, then we set *T*_*D*_*i*__ to be the maximum value of the negative distribution. If these intervals overlap, then we let *μ* and *σ* be the mean and the standard deviation of the positive distribution of SVM probabilities for *D*_*i*_, and we set *T*_*D*_*i*__ = *μ* − 2*σ*.

This set of parameters is used by default in all analysis realized with CLADE. Note that also CLADE_*BEv*_, the version of CLADE that does not include the SVM filter and that considers a score system based on best E-values only, employs domain specific cut-offs. These cut-offs are not the same cut-offs used in CLADE, but they are computed based on E-values of positive and negative training sets. CLADE_*BEv*_-no-cut-off is CLADE_*BEv*_ but uses a unique cut-off of 1*e*-3 for all domains.

Finally, the DAMA software [27] uses the pre-computed list of domain pairs presenting strong co-occurrence in known domain architectures and the list of domain architectures extracted from UniProtKB. DAMA has been run with default parameters. Notice that domains participating in architectures identified by DAMA have E-values < 1*e* − 3 and their length covers at least 40% of the original domain length. Hence, CLADE and CLADE_*BEv*_ architectures contain domains with these features. We stress that DAMA favors the identification of architectures that are made of co-occurring domains, but enriches them with new domains having an E-value < 1*e*-10. These new domains added to an architecture do not co-occur with the existing ones. Hence, CLADE and CLADE_*BEv*_ architectures are made of domains that might co-occur but not necessarily.

### Experiment on SCOP datasets

To realize the comparison experiment on the three SCOP datasets, we used a leave-one-out strategy as follows. Given a domain family *F*_*D*_ in one of the ASTRAL datasets, we considered the set of *n* sequences, coming from different species and associated to *F*_*D*_ in ASTRAL, to create *n* test-sets for *F*_*D*_. Each test-set takes *n* − 1 sequences for training and leaves one sequence out for the test. Then, we tested whether the sequence that was left out could be annotated by a model (or models) constructed without using it, and counted the correct identification of the domain as a true positive (TP), the identification of an erroneous domain as a false positive (FP) and the identification of no domain as a false negative (FN). Note that a domain is “correctly” identified when it belongs to the domain family *F*_*D*_.

This same procedure, was implemented for all systems we wanted to compare: HMMScan, HHblits, CLADE, CLADE_*BEv*_, CLADE_HHblits, CLADE_*BEv*_HHblits.

For HMMScan (run with default parameters), each test-set of *n* − 1 training sequences was first aligned with Clustal W (version 2.1) [65, 66]. Then we used hmmbuild to construct a probabilistic model (a profile) from the alignment. To compare profiles and test sequences we used hmmsearch that employs a composition bias filter by default for eliminating false positives. All tools can be found in the HMMer package—version 3.

For HHblits (run with default parameters), we also constructed a profile from each test-set of *n* − 1 aligned sequences (it is the same alignment as for the HMMScan experiment) but using hhmake. In HHblits experiment, test sequences must be represented by profiles and we used hhblits on HHdb (the HHblits database) to construct them. Profiles were compared with hhsearch [42]. All tools can be found in the HH-suite package—version 2.0.15.

For CLADE, we considered the profile constructed from the test-set of *n* − 1 aligned sequences in the HMMScan experiment, and we constructed additional profiles, one for each sequence in the *n* − 1 set. This was done by using PSI-BLAST and the NR database (see details in Methods). To combine the outputs of the *n* profiles, we trained a SVM by using as positive set the same *n* − 1 sequences and as negative set other sequences never used in training nor test. The negative sequences were chosen randomly into the same SCOP dataset of the positive ones, and by avoiding sequences sharing the same SCOP fold. The SVM was trained with an equal number of positive and negative sequences. The third step of CLADE, handling architectures with DAMA, was not used.

For CLADE_*BEv*_, the version of CLADE that does not include the SVM filter and that considers a score system based on best E-values only, the procedure is the same as for CLADE. The domain-specific cut-offs were learned based on E-values of positive and negative training sets.

For CLADE_HHblits, we carried out the same profile construction as for CLADE, but we replaced PSI-BLAST and HMMScan profiles by HHblits models. Like before, we trained a SVM for combining profile outputs. The only difference is that we used profile-profile comparison to generate the meta-features for training the SVM instead of using sequence-profile comparison like in CLADE. The domain specific cut-offs used in CLADE were computed for the HHblits models using the same learning procedure as that employed for CLADE.

For CLADE_*BEv*_HHblits, the procedure is the same as for CLADE_HHblits, with cut-offs computed for HHblits models that are domain specific but based on E-values (as done for CLADE_*BEv*_).

### Estimated False Discovery Rate for domains identified in protein sequences

Estimating the number of false predictions is an essential step for evaluating the performance of domain identification methods. The basic principle is to estimate the probability that a potential domain has been randomly predicted. We computed the False Discovery Rate (FDR) in two different ways, based on different random models of sequence generation. The key idea is that domain predictions on random sequences arise by chance alone, that predictions on real sequences give us the total number of predictions (true or false), and that their ratio approximate the false discovery rate. For evaluating CLADE (but also HMMScan and DAMA), we run it on real sequences concatenated to its reshuffled ones. The first random model takes a protein and generates 20 different reshuffling of the protein sequence producing new sequences that have the same residue content of the original one and the same length. We call this model “1-mer”. The second random model takes a protein and generates 20 different reshuffling of *k*-mers in the original sequence, for *k* = 4. We call this model “4-mer”. The idea behind this last model is that small *k*-mers within a protein sequence might be more likely to occur than random *k*-mers, since protein sequences might contain repetitive patterns (for instance, blocks of hydrophobic amino-acids or other compositional biases). Given the original set of protein sequences *P* and its associated shuffled sequences *S*, let *P* + *S* be the set of concatenated sequences. Note that the set *P* + *S* is a set containing 20 times more sequences than *P* because from each sequence in *P*, we generated 20 sequences. Then, we computed the number of CLADE domain predictions within the *P*-portion (saying *R*) and the number of predictions within the *S*-portion (saying *A*) of the *P* + *S* sequences, and set the FDR = *A*/*R* for the dataset. This calculation is repeated 20 times, with respect to 20 different reshuffling and the FDR for the 1-mer experiment is considered to be the average of the FDRs of the 20 datasets. The same for the 4-mer experiment. The same strategy was used in [26, 27] (see section “FDR curves” in Methods).

The random reshuffling was realized with the perl function List::Util::shuffle().

#### FDR curves

The FDR can be controlled by modulating the E-value threshold used to filter potential domains [26] and this possibility is used here to compare CLADE, DAMA, HMMScan and HHblits performance. To construct the FDR curves, we followed two strategies. The first strategy [27] generates all domain hits of E-value < 1*e*-3 with HMMER 3.0 and uses them as input for HMMScan and DAMA. For HHblits, it generates domain hits with HHsearch (by matching models, constructed with hhmake from Pfam seed alignments, to profiles of query sequences, constructed with hhblits on HHdb) and considers only those with E-value < 1*e*-3. For CLADE, it generates domain hits with CCMs and SCMs, and, again, considers only those with E-value < 1*e*-3. Each tool reconstructs all architectures at once from the set of their corresponding domain hits. For HHMScan and HHblits, architectures are constructed with Pfam strategy for which, in case of overlapping hits, the hit with better score is chosen. For each tool, points in the corresponding FDR curve are obtained by varying the E-value threshold *M* and filtering out from the architectures all domain hits with E-values > *M*. We varied E-values from 1*e*-60 to 1*e*-3 by small steps. From the resulting set of architectures (made of domain hits of E-value < *M* only), we compute the FDR and the number of domains per protein, and repeat the procedure until the whole curve is drawn.

The interest of this first strategy to generate FDR curves is twofold. When predictions of protein domain architectures are realized over large sets of proteins, we might be interested to have only one run that accepts domains with a high E-value and then decide how to select architectures out of this run, depending on the characteristics of the output we find. Also, we might be interested to explore the landscape of architecture predictions before deciding what E-values to use as a threshold. In doing this, one would like to know whether the tool remains robust while competing with a larger number of domain hits/false positives. This strategy evaluates this aspect of the tools performance.

The second strategy has been presented in [26]. Namely, for each tool, we consider several input sets of domain hits with a E-value threshold < *M*, for different thresholds *M*. They are produced by HMMER 3.0 for HMMScan and DAMA, by HHsearch (as explained for the first FDR strategy) for HHblits, and by HMMER and PSI-BLAST for CLADE. Then, we run each tool on each corresponding set and compute FDR and number of domains per protein for the resulting sets of architectures. This calculation allows constructing curves where the number of domains per proteins is a function of the FDR values. Best performing methods present higher curves. The FDR curves of all methods were computed by using the same set of shuffled sequences. Note that HMMScan and HHblits architectures are obtained by resolving overlaps with a preference for hits of lowest E-value. The curves obtained with this strategy are reported in S3 Fig.

### Evaluation of CLADE, CLADE_BEv_, HMMScan and HHblits performance

The performances of CLADE, CLADE_**BEv**_ (a version without the SVM filter, introduced in the second step of CLADE, and considering a score system based on best E-values only), HMMScan [33] and HHblits [17, 42] have been evaluated by using two standard measures: positive predictive value *PPV* = *TP*/(*TP* + *FP*) (also called precision) and sensitivity *Sen* = *TP*/(*TP* + *FN*) (also known as recall), where *TP*, *FP* and *FN* are true positives, false positives and false negatives, respectively. To give the overall performance for each method, we computed the *F*-score (also called *F*-measure) combining *PPV* and *Sen*, and defined as *F*-score = 2 * *PPV* * *Sen*/(*PPV* + *Sen*). The *F*-score can be interpreted as the harmonic mean of *PPV* and *Sen*, reaching its best value at 1 and worst score at 0. *TP*, *FP* and *FN* are defined as follows: let *s* be a protein sequence, *A* be its domain architecture and *T* be the evaluated method; a true positive is a domain in *A* that is correctly predicted by *T*, a false positive is a domain detected by *T* that overlaps a different domain in *A*, and a false negative is a domain in *A* that is not detected by *T*. The method *T* can detect other domains along *s* that do not overlap domains in *A*, and we shall refer to them as “additional” domains.

### Time complexity of the system and construction of the CLADE models library

CLADE is a pipeline that involves several different tools, and its formal time complexity is hard to establish. The model construction step takes relatively long time due to the time of generation of more than 2 millions (2 389 235) models defining the CLADE library. Namely, each domain construction takes about 30 minutes, and the overall model construction step can be realised in about 3, 7 months on 250 CPUs. Once the models are constructed, domain identification is fast (that is, less than an hour on 100 CPUs for about 5000 proteins).

### Data and software availability

CLADE software and the entire library of CCMs used for the applications presented in this article are available at the address: http://www.lcqb.upmc.fr/CLADE. The CCMs that we generated can be used for annotating any genome and they avoid running the first step of CLADE again. For *P. falciparum*, CLADE website provides access to a downloadable xls file containing the full list of annotations for the 5542 *P. falciparum* proteins (AllDomains.xls). The file also contains the annotations obtained with HHblits and HMMScan, and for each hit, it reports its position, the PlasmoDb accession number, the Pfam domain name, the Pfam clan (if any), and the E-value. The list of disagreeing hits between CLADE and HHblits/HMMScan is also given. A HHblits/HMMScan hit disagrees with CLADE for two reasons: 1. either the hit does not overlap CLADE hits, 2. or the hit overlaps (with an overlapping of any size) a CLADE hit of a different domain and a different clan.

## Supporting Information

S1 Table Reference clades used for building CCMs in CLADE. (PDF)

S2 Table FDR for domain predictions: Comparison between CLADE and HMMScan tested on the two H0 hypotheses. FDR computed for randomly generated domains under two H0 hypothesis tests (see the dataset description in section “Estimated False Discovery Rate for domains identified in protein sequences” in Methods). Mean and variance over 20 randomly generated sets of amino-acids sequences of false domain predictions realized by CLADE and HMMScan. FDR values have been computed by considering that either a domain (All) or the original domain (Original), or a new domain (New) identified on these randomly generated sequences is a false positive. Each set of random sequences is constructed from the 14 831 Pfam_27_ domain families by reshuffling either amino-acids (1-mers) or quadruplets of amino-acids (4-mers).
(PDF)

S3 Table Multi-domain proteins on *P. falciparum* sequences. For different E-value ranges we report the number of proteins predicted by CLADE as having at least two domains (MDP), possibly two occurrences of the same domain. Also, we report the number of multi-domain proteins that were predicted through local models coming from different clades (MDP DC). Notice that for these proteins, at least one prediction was obtained with a clade different of Alveolata. Multi-domain proteins are counted according to the E-value of their higher confidence domain.
(PDF)

S4 Table Improvements and agreement of CLADE over HMMScan domain predictions on *P. falciparum* sequences. Based on the lists of domain predictions provided by CLADE (based on SVM) and CLADE_**BEv**_(based on best E-value), we report the percentage improvement, agreement on domain predictions and agreement on domain architectures of these systems over HMMscan on the full set of *P. falciparum* protein sequences. The improvement has been computed as *X* − *Y*/*Y*, where *X* is the total number of domains predicted by CLADE or CLADE_**BEv**_ and *Y* is the total number of domains predicted by HMMscan with a GA cut-off. Percentage of agreement on domain predictions is the proportion of HMMscan domain predictions shared with CLADE/CLADE_**BEv**_. Percentage of agreement on domain architectures is the proportion of HMMscan domain architectures shared with CLADE/CLADE_**BEv**_. When CLADE and HMMScan annotate a sequence with two domains belonging to the same Pfam clan, we say that the two systems agree.
(PDF)

S5 Table CLADE and HHblits predictions on *P. falciparum* sequences at various FDRs. CLADE is run with an optimal FDR threshold set at 0.1% (top). The values reported on the two CLADE columns (top), correspond to the values in Table 2 for an E-value equal to 1*e*-3. HHblits has been evaluated at different FDR thresholds (middle) and its predictions are compared to CLADE predictions (bottom). In the bottom table we report the total number (Total) of HHblits predictions at FDR 0.1%, 1%, 5%, 10% and how many predictions have been found by HHblits that were obtained by CLADE with an FDR of 0.1% (Shared). Note that, a domain is “shared” by CLADE and HHblits if the two associated domain hits overlap and they belong to the same clan. No condition on the size of the overlapping region is imposed.
(PDF)

S1 Fig Pfam and CLADE flowchart for domain identification. Pfam methodology is showed in solid lines, while modifications proposed by CLADE are shown in dotted lines. For clarity, we duplicated the dataset of Pfam domains to highlight the different use, for model constructions, of the two Pfam sets of sequences SEED (left) and FULL (right). In fact, given a domain *D*^*i*^, the two methodologies make a different use of domain sequences. Based on the SEED set of sequences *S*^i*^ of *D*^*i*^, Pfam produces a multiple sequence alignment, MSA^i*^, and builds a profile hidden Markov model, SCM^i*^. Based on this model, Pfam searches for new homologous sequences and constructs a set of new representative members of *D*^*i*^, called FULL. FULL contains SEED. On the other hand, CLADE builds models selecting sequences in the FULL set *S*^*i*^ according to a reference set of species. These selected sequences are used as queries for building local models (CCM^*i*^) by using PSI-BLAST and the nr database. As a result, CLADE produces a large model library containing both SCMs and CCMs, and uses it to scan genomes to be annotated, like the *P. falciparum* genome (PlasmoDB).
(PDF)

S2 Fig A meta-classifier to combine model predictions. Flow-chart describing the final decision making process on the outputs produced by the models of a domain *D*^*i*^. A meta-classifier (SVM) is trained with five meta-features that are obtained by pre-processing the outputs of the probabilistic models (SCMs and CCMs).
(PDF)

S3 Fig CLADE predicts more domains over a range of FDRs. The y-axis is the number of predicted domains per protein (“signal”), while the x-axis is the FDR (“noise”), so better performing methods have higher curves (more signal for a given noise threshold). CLADE (red) outperforms HMMScan (black), HHblits (green) and DAMA (pink) on the two datasets, 1-mer (top) and 4-mer (bottom), obtained by randomly reshuffling *P. falciparum* sequences (see text). CLADE has been tested under several restrictions and the resulting FDR curves have been added to the plot: CLADE_*ALV*_ (grey), CLADE_*BEv*_ (blue) and CLADE_*BEv*_-no-cut-off (orange). The inset plot zooms the curves on small FDR values (< 0.001). Compare with the plots in Fig 6C: here, the same data are plot with the strategy introduced in [26] and described in Methods.
(PDF)

S4 Fig CLADE on Pfam_27_: Distribution of new domains detected by CLADE. On Pfam_27_, CLADE identifies 2116 new predictions compared to HMMscan. These are domains that either do not overlap with HMMScan hits or they overlap with some hit that is neither the same domain nor the same clan. CLADE predicts 32.21% of the new domains by using CCMs defined from Alveolata species, and 67.79% by using CCMs defined from other clades. Notice that 291 predictions come from CCMs defined from Viruses, Bacteria and Archaea species.
(PDF)
