# Supplementary material for: Improvement in Protein Domain Identification Is Reached by Breaking Consensus, with the Agreement of Many Profiles and Domain Co-occurrence
Source: PLoS Comput Biol. 2016 Jul 29;12(7):e1005038. doi: 10.1371/journal.pcbi.1005038 (PMC4966962; doi:10.1371/journal.pcbi.1005038)
Supplement: S1 Table — (PDF) [file pcbi.1005038.s001.pdf]

Bacteria	Archaea	Viruses	Eukaryota
Actinobacteria	Aenigmarchaeota	Deltavirus	Metazoa
Aquificae	Crenarchaeota	dsDNA viruses, no RNA stage	Fungi
Armatimonadetes	environmental samples	dsRNA viruses	Viridiplantae
Bacteroidetes/Chlorobi group	Euryarchaeota	environmental samples	Alveolata
Caldiserica	Geoarchaeota	Retro-transcribing viruses	Stramenopiles
Chlamydiae/Verrucomicrobia group	Korarchaeota	Satellites	Amoebozoa
Chloroflexi	Nanohaloarchaeota	ssDNA viruses	Euglenozoa
Chrysiogenetes	Parvarchaeota	ssRNA viruses	Choanoflagellida
Cyanobacteria	Thaumarchaeota	unassigned viruses	Fornicata
Deferribacteres	unclassified Archaea	unclassified archaeal viruses	Heterolobosea
Deinococcus-Thermus	Diapherotrites	unclassified phages	Rhodophyta
Dictyoglomi	Nanoarchaeota	unclassified virophages	Parabasalia
Elusimicrobia		unclassified viruses	Opisthokonta
environmental samples			Cryptophyta
Firmicutes			Aquificaceae
Fusobacteria			Rhizaria
Gemmatimonadetes			Haptophyceae
Nitrospinae			Metagenomes
Nitrospirae			
Planctomycetes			
Proteobacteria			
Spirochaetes			
Synergistetes			
Tenericutes			
Thermodesulfobacteria			
Thermotogae			
Fibrobacteres/Acidobacteria group			
unclassified Bacteria			
