# Supplementary material for: Improvement in Protein Domain Identification Is Reached by Breaking Consensus, with the Agreement of Many Profiles and Domain Co-occurrence
Source: PLoS Comput Biol. 2016 Jul 29;12(7):e1005038. doi: 10.1371/journal.pcbi.1005038 (PMC4966962; doi:10.1371/journal.pcbi.1005038)
Supplement: S2 Table — FDR computed for randomly generated domains under two H0 hypothesis tests (see the dataset description in section “Estimated False Discovery Rate for domains identified in protein sequences” in Methods). Mean and variance over 20 randomly generated sets of amino-acids sequences of false domain predictions realized by CLADE and HMMScan. FDR values have been computed by considering that either a domain (All) or the original domain (Original), or a new domain (New) identified on these randomly generated sequences is a false positive. Each set of random sequences is constructed from the 14 831 Pfam27 domain families by reshuffling either amino-acids (1-mers) or quadruplets of amino-acids (4-mers). (PDF) [file pcbi.1005038.s002.pdf]

	1-mer					
	Original		New		All	
	mean	var	mean	var	mean	var
CLADE	0.0003	2.03e-8	0.00698	7.43e-7	0.00728	6.59e-7
HMMScan	0	0	0.0002	3.57e-9	0.0002	3.57e-9
	4-mer					
	Original		New		All	
CLADE	0.023	1.3e-6	0.0076	4.47e-7	0.010	9.32e-7
HMMScan	0.007	4.4e-7	0.0002	1.07e-8	0.007	4.89e-7
