# Supplementary material for: Improvement in Protein Domain Identification Is Reached by Breaking Consensus, with the Agreement of Many Profiles and Domain Co-occurrence
Source: PLoS Comput Biol. 2016 Jul 29;12(7):e1005038. doi: 10.1371/journal.pcbi.1005038 (PMC4966962; doi:10.1371/journal.pcbi.1005038)
Supplement: S3 Table — For different E-value ranges we report the number of proteins predicted by CLADE as having at least two domains (MDP), possibly two occurrences of the same domain. Also, we report the number of multi-domain proteins that were predicted through local models coming from different clades (MDP DC). Notice that for these proteins, at least one prediction was obtained with a clade different of Alveolata. Multi-domain proteins are counted according to the E-value of their higher confidence domain. (PDF) [file pcbi.1005038.s003.pdf]

**E-values range**	**MDP**	**MDP DC**
≤1e-60	60	28
1e-60 < Ev ≤1e-30	237	101
1e-30 < Ev ≤ 1e-15	455	218
1e-15 < Ev ≤ 1e-05	627	376
1e-05 < Ev ≤ 1e-03	309	177
Total	1688	900
