# Supplementary material for: Improvement in Protein Domain Identification Is Reached by Breaking Consensus, with the Agreement of Many Profiles and Domain Co-occurrence
Source: PLoS Comput Biol. 2016 Jul 29;12(7):e1005038. doi: 10.1371/journal.pcbi.1005038 (PMC4966962; doi:10.1371/journal.pcbi.1005038)
Supplement: S4 Table — Based on the lists of domain predictions provided by CLADE (based on SVM) and CLADEBEv(based on best E-value), we report the percentage improvement, agreement on domain predictions and agreement on domain architectures of these systems over HMMscan on the full set of P. falciparum protein sequences. The improvement has been computed as X − Y/Y, where X is the total number of domains predicted by CLADE or CLADEBEv and Y is the total number of domains predicted by HMMscan with a GA cut-off. Percentage of agreement on domain predictions is the proportion of HMMscan domain predictions shared with CLADE/CLADEBEv. Percentage of agreement on domain architectures is the proportion of HMMscan domain architectures shared with CLADE/CLADEBEv. When CLADE and HMMScan annotate a sequence with two domains belonging to the same Pfam clan, we say that the two systems agree. (PDF) [file pcbi.1005038.s004.pdf]

	**CLADE**	**$CLADE_{BEv}$**
**Improvement**	29.88	10.27
**Agreement on domain predictions**	97.54	93.91
**Agreement on domain architectures**	96.94	87.24
