# Supplementary material for: Improvement in Protein Domain Identification Is Reached by Breaking Consensus, with the Agreement of Many Profiles and Domain Co-occurrence
Source: PLoS Comput Biol. 2016 Jul 29;12(7):e1005038. doi: 10.1371/journal.pcbi.1005038 (PMC4966962; doi:10.1371/journal.pcbi.1005038)
Supplement: S5 Table — CLADE is run with an optimal FDR threshold set at 0.1% (top). The values reported on the two CLADE columns (top), correspond to the values in Table 2 for an E-value equal to 1e-3. HHblits has been evaluated at different FDR thresholds (middle) and its predictions are compared to CLADE predictions (bottom). In the bottom table we report the total number (Total) of HHblits predictions at FDR 0.1%, 1%, 5%, 10% and how many predictions have been found by HHblits that were obtained by CLADE with an FDR of 0.1% (Shared). Note that, a domain is “shared” by CLADE and HHblits if the two associated domain hits overlap and they belong to the same clan. No condition on the size of the overlapping region is imposed. (PDF) [file pcbi.1005038.s005.pdf]

CLADE and HHblits at FDR 0.1%

	CLADE		HHblits	
	Cooc	Total	Cooc	Total
Domains occurring on proteins predicted for the first time	467	916	96	269
Domains enriching known protein architectures	1052	1200	327	371
Brand-new domains in *P. falciparum* annotation	603	971	114	195

HHblits predictions

	FDR 1%		FDR 5%		FDR 10%	
	Cooc	Total	Cooc	Total	Cooc	Total
Domains occurring on proteins predicted for the first time	365	824	688	2305	772	3260
Domains enriching known protein architectures	867	1013	1275	2032	1371	2570
Brand-new domains in *P. falciparum* annotation	451	787	902	2646	1025	3869

HHblits vs CLADE predictions

	FDR 0.1%		FDR 1%		FDR 5%		FDR 10%	
	Total	Shared	Total	Shared	Total	Shared	Total	Shared
Domains occurring on proteins predicted for the first time	269	205	824	385	2305	466	3260	477
Domains enriching known protein architectures	371	236	1013	461	2032	524	2570	529
Brand-new domains in *P. falciparum* annotation	195	121	787	247	2646	309	3869	322
