# Supplementary figures and images for: Improvement in Protein Domain Identification Is Reached by Breaking Consensus, with the Agreement of Many Profiles and Domain Co-occurrence
Source: PLoS Comput Biol. 2016 Jul 29;12(7):e1005038. doi: 10.1371/journal.pcbi.1005038 (PMC4966962; doi:10.1371/journal.pcbi.1005038)

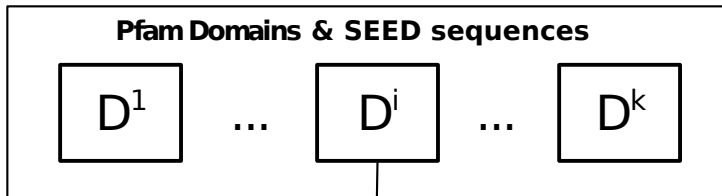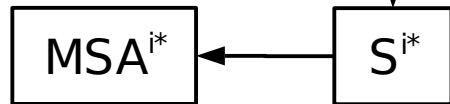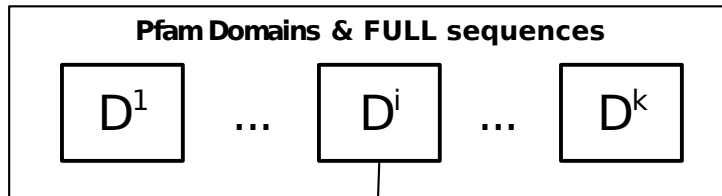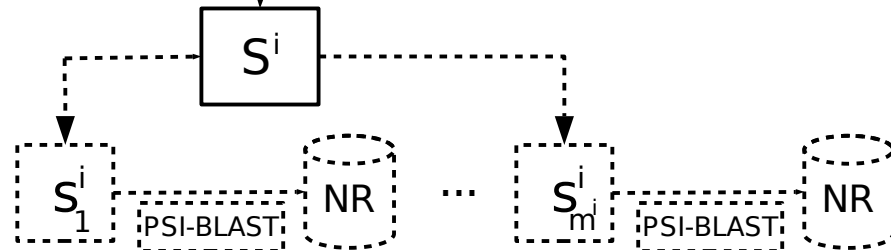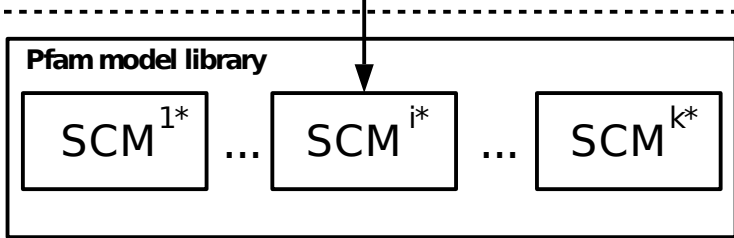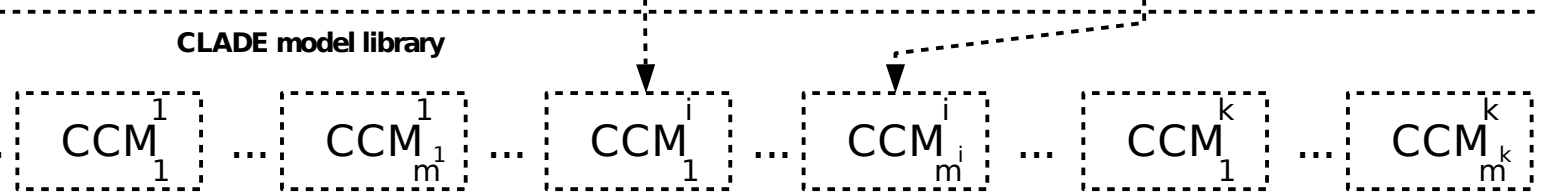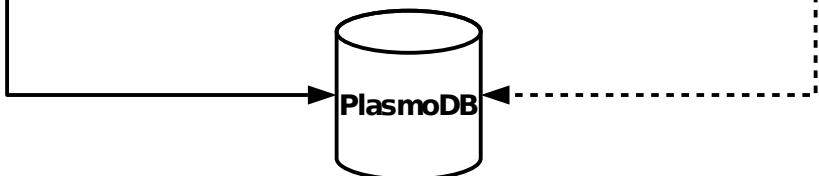

Supplement: S1 Fig — Pfam methodology is showed in solid lines, while modifications proposed by CLADE are shown in dotted lines. For clarity, we duplicated the dataset of Pfam domains to highlight the different use, for model constructions, of the two Pfam sets of sequences SEED (left) and FULL (right). In fact, given a domain Di, the two methodologies make a different use of domain sequences. Based on the SEED set of sequences Si* of Di, Pfam produces a multiple sequence alignment, MSAi*, and builds a profile hidden Markov model, SCMi*. Based on this model, Pfam searches for new homologous sequences and constructs a set of new representative members of Di, called FULL. FULL contains SEED. On the other hand, CLADE builds models selecting sequences in the FULL set Si according to a reference set of species. These selected sequences are used as queries for building local models (CCMi) by using PSI-BLAST and the nr database. As a result, CLADE produces a large model library containing both SCMs and CCMs, and uses it to scan genomes to be annotated, like the P. falciparum genome (PlasmoDB). (PDF) [file pcbi.1005038.s006.pdf]

**Base models**

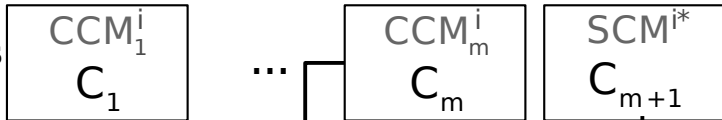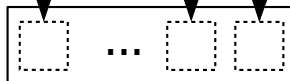

Model outputs

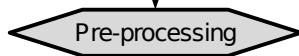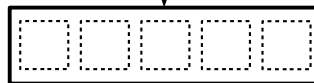

Meta-features

**Meta-classifier**

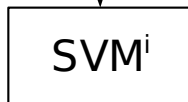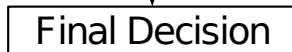

Supplement: S2 Fig — Flow-chart describing the final decision making process on the outputs produced by the models of a domain Di. A meta-classifier (SVM) is trained with five meta-features that are obtained by pre-processing the outputs of the probabilistic models (SCMs and CCMs). (PDF) [file pcbi.1005038.s007.pdf]

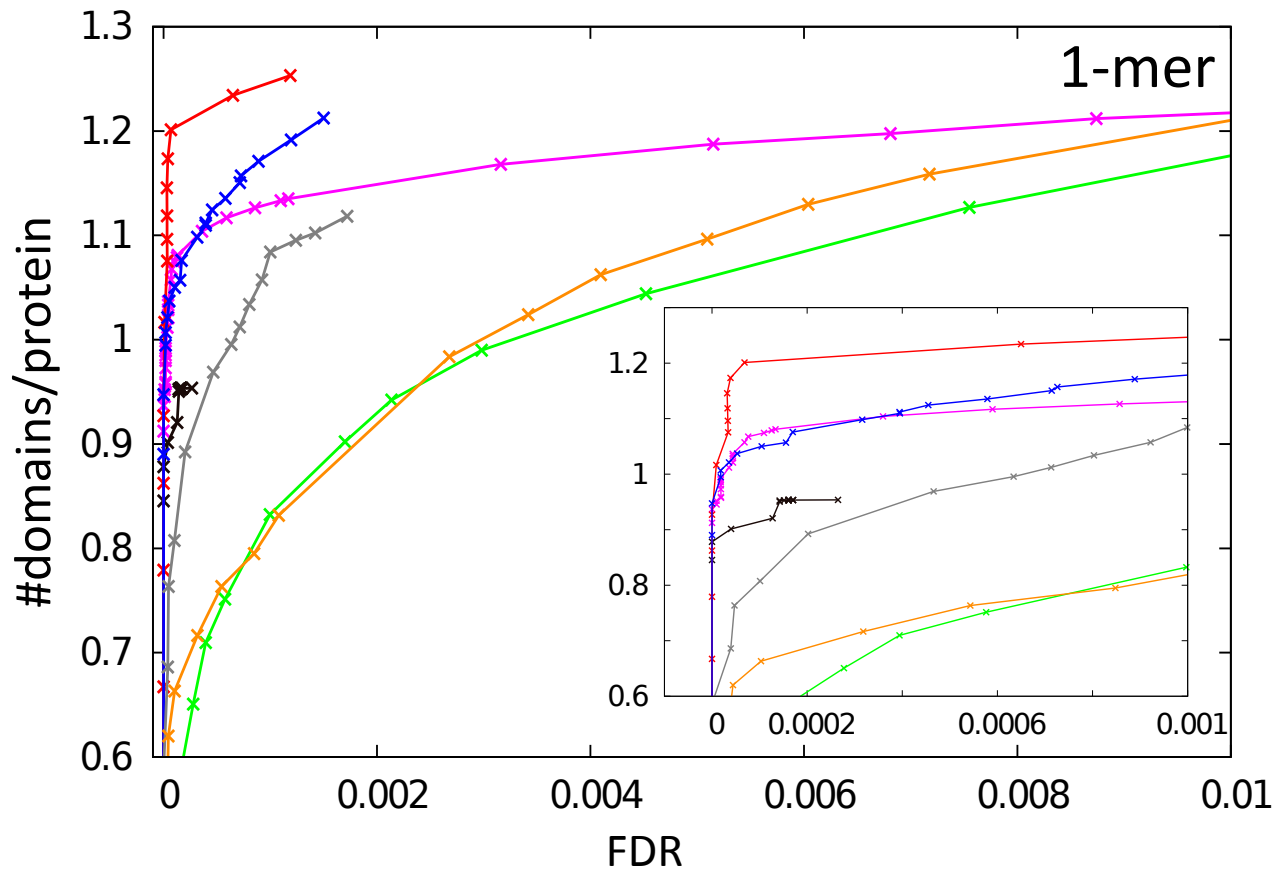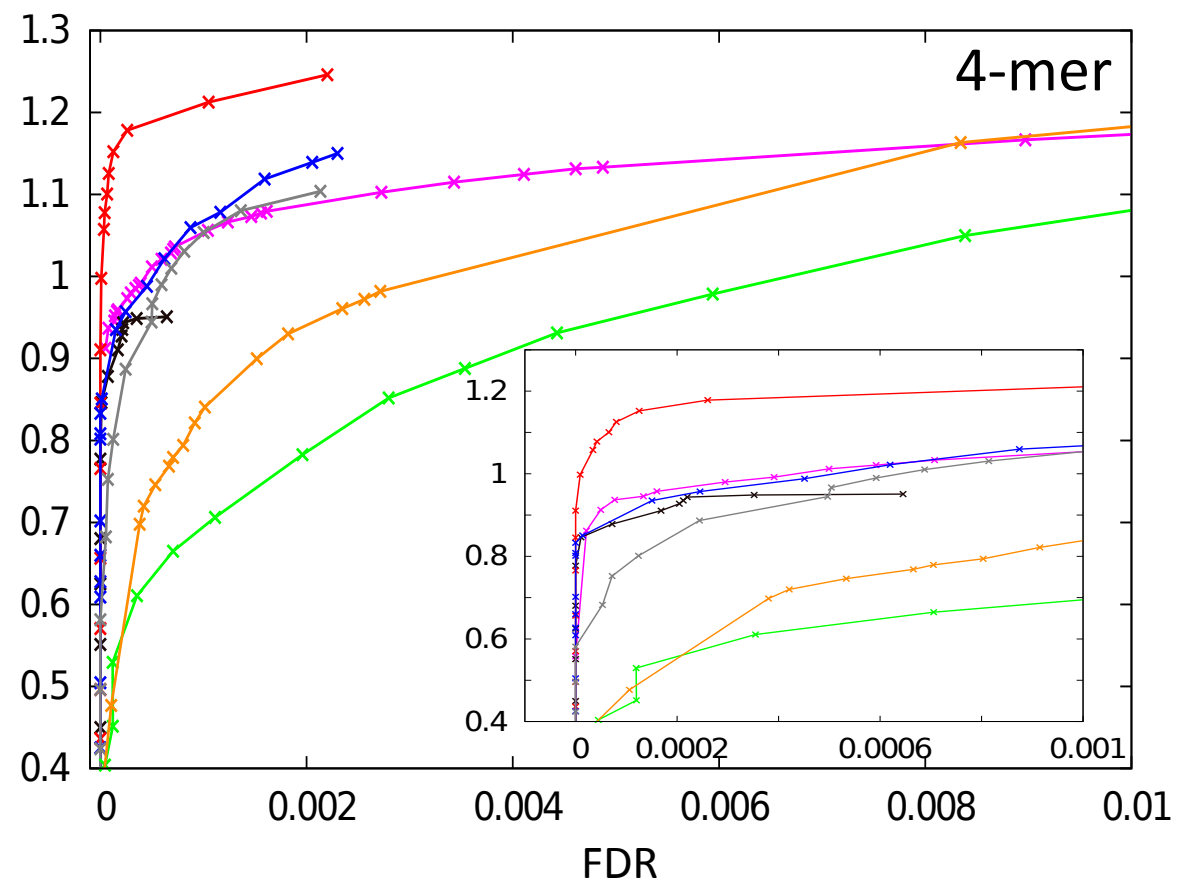

Supplement: S3 Fig — The y-axis is the number of predicted domains per protein (“signal”), while the x-axis is the FDR (“noise”), so better performing methods have higher curves (more signal for a given noise threshold). CLADE (red) outperforms HMMScan (black), HHblits (green) and DAMA (pink) on the two datasets, 1-mer (top) and 4-mer (bottom), obtained by randomly reshuffling P. falciparum sequences (see text). CLADE has been tested under several restrictions and the resulting FDR curves have been added to the plot: CLADEALV (grey), CLADEBEv (blue) and CLADEBEv-no-cut-off (orange). The inset plot zooms the curves on small FDR values (< 0.001). Compare with the plots in Fig 6C: here, the same data are plot with the strategy introduced in [26] and described in Methods. (PDF) [file pcbi.1005038.s008.pdf]

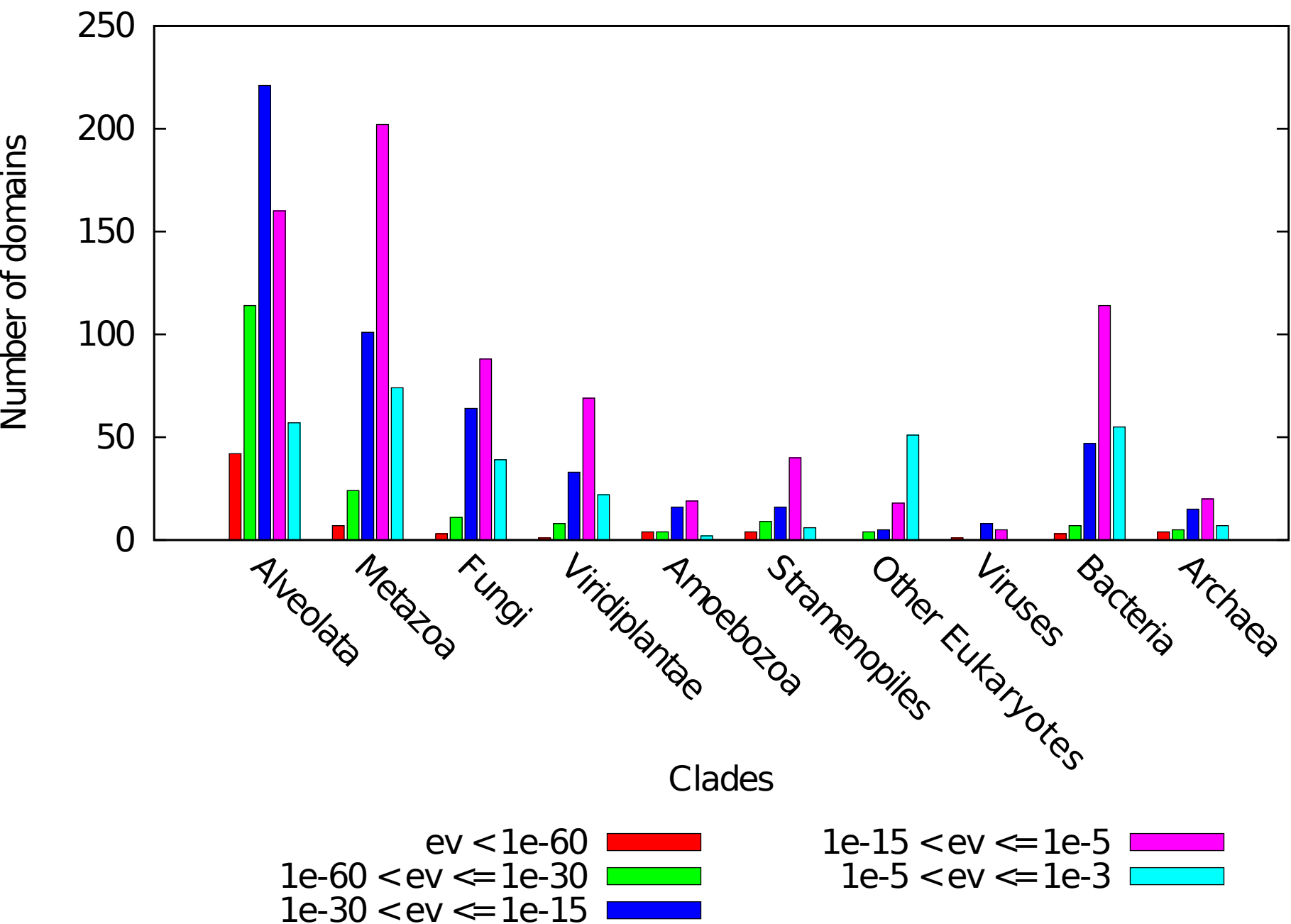

Supplement: S4 Fig — On Pfam27, CLADE identifies 2116 new predictions compared to HMMscan. These are domains that either do not overlap with HMMScan hits or they overlap with some hit that is neither the same domain nor the same clan. CLADE predicts 32.21% of the new domains by using CCMs defined from Alveolata species, and 67.79% by using CCMs defined from other clades. Notice that 291 predictions come from CCMs defined from Viruses, Bacteria and Archaea species. (PDF) [file pcbi.1005038.s009.pdf]
